# Supplementary material for: Which urban land covers/uses are associated with residents’ mortality? A cross-sectional, ecological, pan-European study of 233 cities
Source: BMJ Open. 2019 Nov 19;9(11):e033623. doi: 10.1136/bmjopen-2019-033623 (PMC6886993; doi:10.1136/bmjopen-2019-033623)
Supplement: Supplementary data [file bmjopen-2019-033623supp001.pdf]

Which urban land covers/uses are associated with residents' mortality? A cross-sectional, ecological, pan-European study of 233 cities.

Supplementary Table 1: European Urban Atlas land-use definitions.

**1. Artificial surfaces**		Buildings, roads, all constructions of infrastructure and other artificially sealed or paved areas.
**1.1**	**Urban fabric**	Built-up areas and their associated land, such as gardens, parks, planted areas and non-surfaced public areas and the infrastructure, if these areas are not suitable to be mapped separately with regard to the minimum mapping unit size.
1.1.1	Continuous urban fabric	Predominant residential use: areas with a high degree of soil sealing, independent of their housing scheme (single family houses or high rise dwellings, city centre or suburb).
1.1.2	Discontinuous urban fabric	Predominant residential usage. Contains more than 20% non-sealed areas, independent of their housing scheme (single family houses or high-rise dwellings, city centre or suburb). The non-sealed areas might be private gardens or common green areas.
1.1.2.1	Discontinuous dense urban fabric	Residential buildings, roads and other artificially surfaced areas (Average degree of soil sealing: > 50 - 80%).
1.1.2.2	Discontinuous medium density urban fabric	Residential buildings, roads and other artificially surfaced areas. The vegetated areas are predominant, but the land is not dedicated to forestry or agriculture (Average degree of soil sealing: > 30 - 50%).
1.1.2.3	Discontinuous low density urban fabric	Residential buildings, roads and other artificially surfaced areas. The vegetated areas are predominant, but the land is not dedicated to forestry or agriculture (Average degree of soil sealing: 10 - 30%).
1.1.2.4	Discontinuous very low density urban fabric	Residential buildings, roads and other artificially surfaced areas. The vegetated areas are predominant, but the land is not dedicated to forestry or agriculture. Example: exclusive residential areas with large gardens (Average degree of soil sealing: <10 %).
1.1.3	Isolated structures	Isolated artificial structures with a residential component, such as (small) individual farm houses and related buildings. The mapping unit will never be surrounded by any urban class other than transportation network.
**1.2**	**Industrial, commercial, public, military, private and transport units**	At least 30% of the ground is covered by artificial surfaces. More than 50% of those artificial surfaces are occupied by buildings and / or artificial structures with non-residential use, i.e. industrial, commercial or transport related uses are dominant.
1.2.1	Industrial, commercial, public, military and private units	Artificial structures (e.g. buildings) or artificial surfaces (e.g. concrete, asphalt, tar, macadam, tarmac or otherwise stabilised surface, e.g. compacted soil, devoid of vegetation), occupy most of the surface.
1.2.2	Road and rail network and associated land	The road and railway network (COTS navigation data) is ingested into the classification database according to the

Olsen JR, *et al. BMJ Open* 2019; 9:e033623. doi: 10.1136/bmjopen-2019-033623

		method given in the Annex within the link noted at the end of this table.
1.2.2.1	Fast transit roads and associated land	Roads defined as "motorways" in the COTS navigation data, and motorway rest and service areas and parking areas, only accessible from the motorways.
1.2.2.2	Other roads and associated land	Roads, crossings, intersections and parking areas, including roundabouts and sealed areas with "road surface".
1.2.2.3	Railways and associated land	Railway facilities including stations, cargo stations and service areas.
1.2.3	Port areas	Administrative area of inland harbours and sea ports. Infrastructure of port areas, including quays, dockyards, transport and storage areas and associated areas.
1.2.4	Airports	Administrative area of airports, mostly fenced. Included are all airport installations: runways, buildings and associated land.
**1.3**	**Mine, dump and construction sites**	Open pit extraction sites (sand, quarries) including water surface. Public, industrial or mine dump sites, raw or liquid wastes, legal or illegal, their protecting dikes and / or vegetation belts and associated land such as service areas.
1.3.3	Construction sites	Spaces under construction or development, soil or bedrock excavations for construction purposes or other earthworks visible in the image.
1.3.4	Land without current use	Areas in the vicinity of artificial surfaces still waiting to be used or re-used.
**1.4**	**Artificial non-agricultural vegetated areas**	Vegetation planted and regularly worked by humans; strongly human-influenced. Sporting facilities as functional units independent of being non-sealed, sealed or built-up.
1.4.1	Green urban areas	Public green areas for predominantly recreational use such as gardens, zoos, parks, castle parks. Suburban natural areas that have become and are managed as urban parks. Forests or green areas extending from the surroundings into urban areas are mapped as green urban areas when at least two sides are bordered by urban areas and structures, and traces of recreational use are visible.
1.4.2	Sports and leisure facilities	All sports and leisure facilities including associated land, whether public or commercially managed: e.g. Theresienwiese (Munich), public arenas for any kind of sports including associated green areas, parking places.
**2. Agricultural + semi-natural + wetland areas**		
2.1	Arable land	Fields under rotation system. Can be non-irrigated or permanently irrigated. Also includes rice fields; Fields laid in fallow are included.
2.2	Permanent crops	Fruit orchards, scattered fruit trees with pasture; Vineyards and their nurseries; Roses; Olive groves; Berries and hop plantations.
2.3	Pasture & natural grassland	Grassland; Pasture and meadow under agricultural use, grazed or mechanically harvested.
2.4	Shrubs and / or herbaceous vegetation	Vegetation cover more than 50%, ground coverage of trees with height > 5 m: < 30%, areas with minor / without

	including transitional woodland	artificial or agricultural influence; Sclerophyllous vegetation; Bushy sclerophyllous vegetation (e.g. maquis, garrigue); Abandoned arable land with bushes; Woodland degradation: storm, snow, insects or air pollution; Areas under power transmission lines inside forest; Fire breaks; Steep bushy slopes of eroded areas; Abandoned vineyards or orchards, arable land and pasture land under natural colonisation; Dehesas with bush proliferation indicating no agricultural or farming use for a rather long time; Bushy areas along creeks.
2.5	Moors and heathland	Bushes, shrubs and herbaceous plants, dwarf forest in alpine or coastal regions (Pinus Mugo forests). Height is maximum 3 m in climax stage.
2.6	Beaches, dunes, sand	< 10% vegetation cover; Beaches, dunes and sand plains, (coastal or inland location), gravel along rivers; Seasonal rivers, if water is characteristic for a shorter part of the year (< 2 months).
2.7	Bare rocks	> 90% of the land surface of bare rocks, (i.e. < 10% vegetation); Rocks, gravel fields, landslides; Scree (fragments resulting from mechanical and chemical erosion. Weathering rocks forming heaps of coarse debris at the foot of steep slopes), cliffs, rocks.
2.8	Sparsely vegetated areas	Steppes, tundra, badlands, scattered high altitude vegetation. Bare soils inside military training areas. Vegetation cover 10 - 50%.
2.9	Burnt areas	Recently burnt forest or shrubs (but not natural grassland), still mainly black on EO data.
2.10	Snow and ice	Glacier and perpetual snow
2.11	Inland wetlands	Areas flooded or liable to flooding during a large part of the year by fresh, brackish or standing water with specific vegetation coverage made of low shrub, semi-ligneous or herbaceous species; Water fringe vegetation, reed beds of lakes, rivers and brooks. Sedge and fen-sedge beds, swamps; Peat bogs, with or without peat extracting areas; Shallow water areas covered with reed; Seasonal rivers, if water course is not visible in the EO data.
2.12	Coastal wetlands	Areas, flooded or liable to flooding during a large part of the year by brackish or saline water, susceptible to flooding by sea water. Often in the process of filling in and gradually being colonised by halophytic plants; Specific vegetation coverage made of low shrub, semi-ligneous or herbaceous species; Alluvial planes, marshes and intertidal flats; Salinas (salt production sites by evaporation).
**3. Forest (natural and plantation)**		With ground coverage of tree canopy > 30%, tree height > 5 m, including bushes and shrubs at the fringe of the forest; Included are plantations such as Populus plantations, Christmas tree plantations; Forest regeneration / re-colonisation: clear cuts, new forest plantations.

**4. Water**	The visible water surface area on the EO data is delineated: Sea; Lakes; Fish ponds (natural, artificial); Rivers, including channelled rivers; Canals.

Adapted from: http://ec.europa.eu/regional_policy/sources/tender/pdf/2012066/annexe2.pdf
