# Supplementary material for: Which urban land covers/uses are associated with residents’ mortality? A cross-sectional, ecological, pan-European study of 233 cities
Source: BMJ Open. 2019 Nov 19;9(11):e033623. doi: 10.1136/bmjopen-2019-033623 (PMC6886993; doi:10.1136/bmjopen-2019-033623)
Supplement: Supplementary data [file bmjopen-2019-033623supp002.pdf]

Which urban land covers/uses are associated with residents' mortality? A cross-sectional, ecological, pan-European study of 233 cities.

Supplementary Table 2: Associations between land cover/use proportion and SMR for Western European cities

Quintiles (proportion of land-use)	Males				Females			
	Coef	p	LL 95% CI	UL 95% CI	Coef	p	LL 95% CI	UL 95% CI
**Agricultural, semi-natural areas, wetlands**								
1 (Least)	ref				ref			
2	-10.22	0.01	-17.69	-2.75	-11.66	0.02	-20.75	-2.58
3	-11.67	0.00	-17.30	-6.04	-13.03	0.02	-23.12	-2.94
4	-12.63	0.00	-19.14	-6.13	-19.71	0.01	-33.78	-5.65
5 (Most)	-13.66	0.00	-21.08	-6.23	-18.32	0.00	-29.77	-6.88
	(Wald test of parameter: 0.006)				*(Wald test of parameter: 0.052)*			
**Airports ***								
1 (Least)	ref				Ref			
2	-2.87	0.29	-8.50	2.76	-3.03	0.46	-11.66	5.61
3	-8.70	0.00	-13.48	-3.93	-10.73	0.02	-19.09	-2.36
4 (Most)	-2.48	0.43	-8.97	4.02	-6.72	0.21	-17.71	4.26
	(Wald test of parameter: 0.005)				*(Wald test of parameter: 0.062)*			
**Construction sites**								
1 (Least)	ref				ref			
2	-3.07	0.37	-10.22	4.07	-9.93	0.13	-23.26	3.40
3	-1.44	0.67	-8.65	5.76	-8.35	0.16	-20.32	3.61
4	-0.68	0.91	-13.98	12.61	-12.36	0.09	-27.14	2.42
5 (Most)	-1.20	0.74	-8.84	6.44	-9.11	0.20	-23.68	5.45
	(Wald test of parameter: 0.814)				*(Wald test of parammeter:0.358)*			
**Continuous urban fabric**								
1 (Least)	ref				ref			
2	1.56	0.60	-4.72	7.84	-5.62	0.10	-12.45	1.21
3	0.00	1.00	-6.03	6.03	-4.66	0.30	-13.98	4.66
4	4.24	0.12	-1.31	9.78	-0.43	0.92	-9.09	8.23
5 (Most)	3.03	0.39	-4.26	10.31	0.09	0.99	-13.58	13.77
	(Wald test of parameter: 0.554)				*(Wald test of parameter: 0.1715)*			
**Discontinuous dense urban fabric**								
1 (Least)	ref				ref			
2	-2.38	0.59	-11.58	6.817	-5.73	0.42	-20.69	9.23
3	-3.39	0.47	-13.27	6.49	-4.45	0.63	-23.80	14.90
4	2.11	0.51	-4.66	8.87	2.28	0.73	-11.88	16.45
5 (Most)	7.36	0.15	-3.02	17.74	9.55	0.26	-7.89	26.99
	(Wald test of parameter: 0.051)				*(Wald test of parameter: 0.093)*			
**Discontinuous medium density urban fabric**								
1 (Least)	ref				ref			

Olsen JR, *et al. BMJ Open* 2019; 9:e033623. doi: 10.1136/bmjopen-2019-033623

2	-0.27	0.95	-8.74	8.19	-7.70	0.19	-19.80	4.39
3	-3.20	0.29	-9.40	2.99	-5.60	0.19	-14.34	3.14
4	-4.87	0.10	-10.89	1.16	-11.30	0.04	-21.67	-0.93
5 (Most)	-3.54	0.38	-12.00	4.91	-13.12	0.01	-22.56	-3.67
	(Wald test of parameter: 0.456)				*(Wald test of parameter: 0.097)*			
**Discontinuous low density urban fabric**								
1 (Least)	ref				ref			
2	0.46	0.89	-6.48	7.40	-3.85	0.41	-13.57	5.87
3	-0.40	0.90	-6.95	6.16	-5.65	0.30	-17.07	5.76
4	-0.58	0.92	-12.46	11.31	-6.61	0.50	-27.00	13.79
5 (Most)	7.98	0.01	2.22	13.75	7.92	0.10	-1.62	17.46
	(Wald test of parameter: 0.005)				*(Wald test of parameter: 0.027)*			
**Discontinuous very low density urban fabric**								
1 (Least)	ref				ref			
2	1.10	0.83	-9.74	11.93	-1.87	0.78	-16.24	12.50
3	-5.35	0.25	-14.90	4.212	-13.21	0.10	-29.21	2.79
4	-5.37	0.16	-13.18	2.45	-13.85	0.01	-24.06	-3.65
5 (Most)	-5.75	0.08	-12.28	0.77	-12.42	0.03	-22.99	-1.85
	(Wald test of parameter: 0.313)				*(Wald test of parameter: 0.034)*			
**Residential proportion**								
1 (Least)	ref				ref			
2	1.13	0.74	-5.96	8.22	0.82	0.90	-13.05	14.68
3	2.77	0.29	-2.63	8.17	0.23	0.96	-9.54	10.00
4	3.87	0.08	-0.53	8.26	3.86	0.11	-0.96	8.68
5 (Most)	13.19	0.00	7.88	18.49	17.39	0.00	9.61	25.18
	(Wald test of parameter: <0.001)				*(Wald test of parameter: 0.004)*			
**Fast transit roads and associated land**								
1 (Least)	ref				ref			
2	-5.44	0.24	-14.87	4.00	-14.64	0.03	-27.59	-1.69
3	-3.46	0.25	-9.66	2.74	-10.80	0.03	-20.54	-1.07
4	-3.61	0.41	-12.81	5.59	-15.33	0.02	-28.11	-2.55
5 (Most)	0.62	0.85	-6.44	7.68	-6.77	0.16	-16.47	2.93
	(Wald test of parameter: 0.722)				*(Wald test of parameter: 0.126)*			
**Forests**								
1 (Least)	ref				ref			
2	-4.60	0.11	-10.36	1.158271	-9.71	0.03	-18.44	-0.97842
3	-4.53	0.13	-10.66	1.600225	-8.48	0.10	-18.81	1.854112
4	-9.54	0.06	-19.44	0.372522	-15.13	0.07	-31.88	1.617823
5 (Most)	-9.03	0.03	-16.99	-1.07548	-12.59	0.05	-25.08	-0.10597
	(Wald test of parameter: 0.1562)				*(Wald test of parameter: 0.0048)*			
**Green urban areas**								
1 (Least)	ref				ref			
2	1.60	0.58	-4.47	7.66	-0.02	1.00	-7.11	7.07
3	3.96	0.18	-2.05	9.96	1.43	0.74	-7.81	10.67
4	7.23	0.06	-0.26	14.73	9.57	0.03	1.21	17.93
5 (Most)	11.33	0.02	2.44	20.21	15.06	0.01	4.45	25.67

	(Wald test of parameter: 0.132)				*(Wald test of parameter: 0.027)*			
**Industrial, commercial, public, military**								
1 (Least)		ref				ref		
2	2.02	0.35	-2.51	6.55	-0.23	0.94	-6.54	6.08
3	1.34	0.67	-5.24	7.93	1.75	0.67	-6.93	10.42
4	3.24	0.22	-2.16	8.65	6.76	0.01	2.31	11.21
5 (Most)	10.64	0.01	3.79	17.50	11.02	0.04	0.93	21.11
	(Wald test of parameter: 0.049)				*(Wald test of parameter: 0.023)*			
**Isolated structures**								
1 (Least)		ref				ref		
2	-5.04	0.16	-12.40	2.32	-9.40	0.07	-19.77	0.97
3	-6.42	0.00	-10.46	-2.39	-10.60	0.02	-18.98	-2.23
4	-7.94	0.02	-14.52	-1.35	-7.23	0.05	-14.35	-0.12
5 (Most)	-7.87	0.04	-15.28	-0.46	-8.46	0.10	-18.68	1.77
	(Wald test of parameter: 0.029)				*(Wald test of parameter: 0.145)*			
**Land without current use**								
1 (Least)		ref				ref		
2	0.65	0.79	-4.57	5.88	-4.16	0.39	-14.34	6.07
3	1.09	0.67	-4.36	6.55	-4.20	0.27	-12.08	3.69
4	0.98	0.67	-3.80	5.77	-1.62	0.43	-5.91	2.67
5 (Most)	10.18	0.05	0.07	20.30	4.58	0.37	-6.12	15.28
	(Wald test of parameter: 0.249)				*(Wald test of parameter: 0.316)*			
**Mineral extraction and dump sites**								
1 (Least)		ref				ref		
2	-3.71	0.22	-9.90	2.47	-6.17	0.11	-13.97	1.63
3	-5.83	0.12	-13.36	1.70	-8.17	0.23	-22.21	5.88
4	-2.74	0.42	-9.85	4.37	-5.45	0.10	-12.10	1.19
5 (Most)	-2.92	0.27	-8.40	2.56	-7.98	0.01	-13.45	-2.52
	(Wald test of parameter: 0.081)				*(Wald test of parameter: 0.061)*			
**Other roads and associated land**								
1 (Least)		ref				ref		
2	-0.95	0.71	-6.38	4.494284	-6.44	0.14	-15.22	2.330186
3	0.64	0.80	-4.66	5.935122	0.46	0.89	-6.43	7.363465
4	2.15	0.39	-3.02	7.322068	4.22	0.15	-1.79	10.23612
5 (Most)	9.49	0.02	1.55	17.42118	6.69	0.17	-3.24	16.61991
	(Wald test of parameter: 0.1889)				*(Wald test of parameter: 0.2091)*			
**Railways and associated land**								
1 (Least)		ref				ref		
2	1.12	0.81	-8.66	10.90	-5.72	0.42	-20.40	8.95
3	2.41	0.31	-2.51	7.31	-2.99	0.66	-17.47	11.49
4	5.82	0.06	-0.21	11.86	-2.74	0.44	-10.10	4.62
5 (Most)	1.81	0.56	-4.71	8.32	-7.73	0.10	-17.10	1.64
	(Wald test of parameter: 0.190)				*(Wald test of parameter: 0.2796)*			
**Sports and leisure facilities**								
1 (Least)		ref				ref		
2	1.68	0.65	-6.25	9.61	-4.11	0.59	-20.19	11.97

3	2.48	0.36	-3.15	8.12	0.01	1.00	-10.95	10.97
4	4.60	0.06	-0.11	9.31	9.67	0.03	1.18	18.16
5 (Most)	10.39	0.02	1.82	18.95	14.12	0.02	2.88	25.35
	(Wald test of parameter: 0.179)				*(Wald test of parameter: 0.086)*			
**Water bodies**								
1 (Least)	ref				ref			
2	5.33	0.18	-2.70	13.36	6.06	0.45	-10.56	22.69
3	-1.35	0.70	-8.79	6.08	2.29	0.68	-9.55	14.12
4	0.31	0.91	-5.31	5.93	4.27	0.34	-4.96	13.51
5 (Most)	-1.60	0.53	-6.98	3.79	2.32	0.66	-8.86	13.51
	(Wald test of parameter: 0.317)				*(Wald test of parameter: 0.467)*			

* Due to low proportion of land area classed as airports within cities, the first two quintiles combined within the model, essentially representing less than ~0.3% of the total area.

Supplementary Table 3: Associations between land cover/use proportion and SMR for Eastern European cities

Quintiles (proportion of land-use)	Males				Females			
	Coef	p	LL 95% CI	UL 95% CI	Coef	p	LL 95% CI	UL 95% CI
**Agricultural, semi-natural areas, wetlands**								
1 (Least)	ref				ref			
2	-14.45	0.07	-30.34	1.44	-3.68	0.36	-12.24	4.88
3	-11.51	0.20	-30.34	7.32	-2.86	0.44	-10.81	5.08
4	-18.67	0.05	-37.03	-0.32	-7.14	0.13	-16.67	2.39
5 (Most)	-3.89	0.71	-26.56	18.78	5.76	0.39	-8.51	20.03
	(Wald test of parameter: 0.017)				*(Wald test of parameter: 0.014)*			
**Airports ***								
1 (Least)	ref				ref			
2	7.86	0.19	-4.52	20.25	8.08	0.05	0.24	15.92
3 (Most)	15.35	0.02	2.44	28.27	8.64	0.01	2.70	14.58
	(Wald test of parameter: 0.047)				*(Wald test of parameter: 0.008)*			
**Construction sites**								
1 (Least)	ref				ref			
2	0.44	0.94	-11.40	12.27177	-3.87	0.19	-9.93	2.201498
3	1.91	0.66	-7.51	11.33426	0.88	0.80	-6.53	8.280001
4	-4.12	0.52	-17.96	9.71732	-5.57	0.26	-15.84	4.707548
5 (Most)	2.19	0.77	-13.76	18.14004	-4.80	0.24	-13.33	3.721612
	(Wald test of parameter: 0.725)				*(Wald test of parameter: 0.368)*			
**Continuous urban fabric**								
1 (Least)	ref				ref			
2	-1.62	0.78	-14.34	11.10	-0.34	0.92	-7.69	7.01

Olsen JR, *et al. BMJ Open* 2019; 9:e033623. doi: 10.1136/bmjopen-2019-033623

3	-4.22	0.59	-20.97	12.53	0.40	0.95	-14.96	15.77
4	-6.72	0.39	-23.49	10.05	-0.36	0.96	-15.72	14.99
5 (Most)	-0.93	0.87	-13.68	11.82	5.78	0.36	-7.69	19.25
	(Wald test of parameter: 0.791)				*(Wald test of parameter: 0.070)*			
**Discontinuous dense urban fabric**								
1 (Least)			ref				ref	
2	-3.51	0.51	-15.00	7.98	-0.71	0.89	-11.37	9.96
3	-2.21	0.64	-12.37	7.96	-6.60	0.13	-15.61	2.42
4	-3.91	0.47	-15.38	7.56	-7.30	0.15	-17.71	3.12
5 (Most)	3.36	0.52	-7.86	14.60	-3.33	0.53	-14.60	7.93
	(Wald test of parameter: 0.737)				*(Wald test of parameter: 0.09)*			
**Discontinuous medium density urban fabric**								
1 (Least)			ref				ref	
2	-2.18	0.64	-12.17	7.80	-2.01	0.50	-8.34	4.32
3	7.49	0.08	-1.13	16.12	7.87	0.01	2.48	13.26
4	2.02	0.74	-11.17	15.21	5.07	0.45	-9.24	19.38
5 (Most)	7.92	0.35	-10.01	25.86	1.53	0.80	-11.36	14.42
	(Wald test of parameter: 0.161)				*(Wald test of parameter: 0.083)*			
**Discontinuous low density urban fabric**								
1 (Least)			ref				ref	
2	0.67	0.92	-14.40	15.73	0.19	0.98	-18.09	18.47
3	2.83	0.63	-9.82	15.48	1.37	0.82	-12.03	14.77
4	5.38	0.39	-8.08	18.84	0.88	0.92	-17.35	19.10
5 (Most)	1.47	0.84	-13.93	16.87	-3.82	0.69	-24.54	16.89
	(Wald test of parameter: 0.822)				*(Wald test of parameter: 0.408)*			
**Discontinuous very low density urban fabric**								
1 (Least)			ref				ref	
2	12.10	0.03	1.90	22.30	3.23	0.30	-3.38	9.85
3 (Most)	0.62	0.91	-10.81	12.06	-0.38	0.93	-9.62	8.85
	(Wald test of parameter: 0.065)				*(Wald test of parameter: 0.563)*			
**Residential proportion**								
1 (Least)			ref				ref	
2	-0.70	0.92	-16.68	15.27	-6.19	0.29	-18.52	6.15
3	-4.58	0.40	-16.14	6.97	-2.68	0.64	-14.96	9.60
4	-2.50	0.61	-13.04	8.04	-5.32	0.21	-14.18	3.54
5 (Most)	3.56	0.66	-13.90	21.02	-0.70	0.90	-12.82	11.42
	(Wald test of parameter: 0.469)				*(Wald test of parameter: 0.155)*			
**Fast transit roads and associated land**								
1 (Least)			ref				ref	
2	3.08	0.61	-9.88	16.04	2.18	0.58	-6.31	10.68
3 (Most)	0.83	0.89	-12.17	13.82	2.81	0.63	-9.79	15.41
	(Wald test of parameter: 0.836)				*(Wald test of parameter: 0.846)*			

Olsen JR, *et al. BMJ Open* 2019; 9:e033623. doi: 10.1136/bmjopen-2019-033623

**Forests**								
1 (Least)		ref				ref		
2	-6.93	0.28	-20.41	6.55	-13.55	0.02	-24.82	-2.28
3	-5.82	0.13	-13.75	2.11	-10.43	0.00	-16.25	-4.62
4	-0.73	0.89	-12.67	11.21	-8.27	0.13	-19.36	2.83
5 (Most)	-4.99	0.20	-13.08	3.10	-15.96	0.00	-24.74	-7.17
	(Wald test of parameter: 0.309)				*(Wald test of parameter: 0.010)*			
**Green urban areas**								
1 (Least)		ref				ref		
2	1.48	0.67	-6.00	8.95	4.07	0.49	-8.47	16.62
3	-5.99	0.06	-12.37	0.39	-3.06	0.50	-12.89	6.77
4	-10.07	0.05	-19.96	-0.19	-9.26	0.17	-23.29	4.77
5 (Most)	15.37	0.02	3.66	27.08	3.85	0.50	-8.48	16.19
	(Wald test of parameter: 0.001)				*(Wald test of parameter: 0.004)*			
**Industrial, commercial, public, military**								
1 (Least)		ref				ref		
2	-12.53	0.14	-29.98	4.91	-0.92	0.91	-18.26	16.43
3	-1.70	0.81	-16.73	13.32	4.72	0.48	-9.75	19.20
4	-7.70	0.22	-20.69	5.30	-1.34	0.80	-12.77	10.08
5 (Most)	-2.81	0.51	-11.94	6.31	6.09	0.06	-0.19	12.37
	(Wald test of parameter: 0.491)				*(Wald test of parameter: 0.032)*			
**Isolated structures**								
1 (Least)		ref				ref		
2	-5.55	0.38	-19.15	8.04	-7.79	0.15	-18.77	3.19
3	-2.28	0.70	-15.09	10.53	-2.19	0.58	-10.70	6.31
4	-5.12	0.33	-16.15	5.92	-10.66	0.01	-17.95	-3.37
5 (Most)	-6.14	0.33	-19.55	7.26	-12.35	0.00	-16.73	-7.98
	(Wald test of parameter: 0.814)				*(Wald test of parameter: 0.001)*			
**Land without current use**								
1 (Least)		ref				ref		
2	0.14	0.96	-6.55	6.84	-2.02	0.43	-7.45	3.41
3	8.29	0.11	-2.13	18.71	11.30	0.01	3.43	19.18
4	14.75	0.01	5.70	23.81	12.81	0.00	6.49	19.14
5 (Most)	13.67	0.04	1.05	26.29	9.30	0.01	3.09	15.51
	(Wald test of parameter: <0.001)				*(Wald test of parameter: <0.001)*			
**Mineral extraction and dump sites**								
1 (Least)		ref				ref		
2	7.67	0.18	-4.27	19.60	5.02	0.22	-3.42	13.46
3	8.34	0.05	-0.16	16.83	6.73	0.04	0.27	13.18
4	7.86	0.14	-3.11	18.84	7.92	0.12	-2.45	18.30

5 (Most)	7.16	0.22	-4.90	19.23	4.74	0.20	-2.91	12.39
	(Wald test of parameter: 0.139)				*(Wald test of parameter: 0.016)*			
**Other roads and associated land**								
1 (Least)	ref				ref			
2	-8.85	0.07	-18.65	0.94	-1.47	0.61	-7.77	4.82
3	-12.31	0.03	-22.79	-1.82	-4.27	0.22	-11.59	3.05
4	-6.65	0.17	-16.66	3.35	1.69	0.42	-2.76	6.15
5 (Most)	1.52	0.82	-13.31	16.35	7.74	0.15	-3.28	18.76
	(Wald test of parameter: 0.080)				*(Wald test of parameter: 0.174)*			
**Railways and associated land**								
1 (Least)	ref				ref			
2	-3.66	0.43	-13.61	6.29	-1.69	0.61	-8.85	5.46
3	-0.19	0.97	-11.74	11.35	3.45	0.42	-5.74	12.65
4	0.12	0.99	-13.95	14.19	3.57	0.52	-8.36	15.49
5 (Most)	-3.42	0.52	-14.82	7.98	4.93	0.27	-4.39	14.26
	(Wald test of parameter: 0.883)				*(Wald test of parameter: 0.215)*			
**Sports and leisure facilities**								
1 (Least)	ref				ref			
2	-7.42	0.17	-18.66	3.82	-7.20	0.13	-16.97	2.58
3	-18.90	0.00	-27.75	-10.06	-15.15	0.04	-29.78	-0.53
4	-13.80	0.02	-25.27	-2.33	-13.98	0.06	-28.92	0.97
5 (Most)	-19.87	0.00	-31.05	-8.68	-15.16	0.06	-31.38	1.07
	(Wald test of parameter: 0.002)				*(Wald test of parameter: 0.259)*			
**Water bodies**								
1 (Least)	ref				ref			
2	1.97	0.53	-4.69	8.63	0.30	0.91	-5.51	6.10
3	3.49	0.35	-4.42	11.41	1.22	0.70	-5.72	8.16
4	9.52	0.10	-2.14	21.18	5.92	0.02	1.18	10.65
5 (Most)	7.52	0.20	-4.67	19.72	1.55	0.54	-3.90	7.00
	(Wald test of parameter: 0.474)				*(Wald test of parameter: 0.062)*			

* Due to low proportion of land area classed as airports within cities, the first three quintiles combined within the model, essentially representing less than ~0.1% of the total area.

Olsen JR, *et al. BMJ Open* 2019; 9:e033623. doi: 10.1136/bmjopen-2019-033623
