# Supplementary material for: Which urban land covers/uses are associated with residents’ mortality? A cross-sectional, ecological, pan-European study of 233 cities
Source: BMJ Open. 2019 Nov 19;9(11):e033623. doi: 10.1136/bmjopen-2019-033623 (PMC6886993; doi:10.1136/bmjopen-2019-033623)
Supplement: Author's manuscript [file bmjopen-2019-033623.draft_revisions.pdf]

BMJ Open is committed to open peer review. As part of this commitment we make the peer review history of every article we publish publicly available.

When an article is published we post the peer reviewers' comments and the authors' responses online. We also post the versions of the paper that were used during peer review. These are the versions that the peer review comments apply to.

The versions of the paper that follow are the versions that were submitted during the peer review process. They are not the versions of record or the final published versions. They should not be cited or distributed as the published version of this manuscript.

BMJ Open is an open access journal and the full, final, typeset and author-corrected version of record of the manuscript is available on our site with no access controls, subscription charges or pay-per-view fees (http://bmjopen.bmj.com).

If you have any questions on BMJ Open's open peer review process please email info.bmjopen@bmj.com

BMJ Open

Which urban land covers/uses are associated with residents' mortality? A cross-sectional, ecological, pan-European study of 233 cities.

Journal:	*BMJ Open*
Manuscript ID	bmjopen-2019-033623
Article Type:	Original research
Date Submitted by the Author:	14-Aug-2019
Complete List of Authors:	Olsen, Jonathan; University of Glasgow, Institute of Health and Wellbeing<br>Nicholls, Natalie; University of Glasgow, MRC/CSO Social and Public Health Sciences Unit<br>Moon, Graham; University of Southampton, Geography and Environment<br>Pearce, Jamie; University of Edinburgh, Institute of Geography and the Lived Environment<br>Shortt, Niamh; University of Edinburgh, Centre for Research on Environment, Society and Health. School of Geosciences<br>Mitchell, Richard; Glasgow University, Public Health
Keywords:	EPIDEMIOLOGY, PUBLIC HEALTH, STATISTICS & RESEARCH METHODS

SCHOLARONE™
Manuscripts

Which urban land covers/uses are associated with residents' mortality? A cross-sectional, ecological, pan-European study of 233 cities.

Jonathan R. Olsen[1], Natalie Nicholls[1], Graham Moon[2], Jamie Pearce[3], Niamh Shortt[3], Richard Mitchell[1]

[1]MRC/CSO Social and Public Health Sciences Unit, University of Glasgow, Glasgow, UK
[2]School of Geography and Environmental Sciences, University of Southampton, Southampton, UK
[3]School of Geosciences, University of Edinburgh, Edinburgh, UK

Correspondence:
Dr Jonathan R Olsen
MRC/CSO Social and Public Health Sciences Unit
University of Glasgow
200 Renfield Street
Glasgow
G2 3AX
Phone: 0141 353 7539
Email: jonathan.olsen@glasgow.ac.uk

Key words: urban; mortality; landscape; ecology; land use; land cover; urban landscape.

Tables : 2
Figures : 3
Supplementary tables : 3

For peer review only

For peer review only - http://bmjopen.bmj.com/site/about/guidelines.xhtml

Abstract

Objectives:

The study aim was to determine whether the range and distribution of all, and proportions of specific, land covers/uses within European cities is associated with the city-specific mortality rate.

Setting:

233 European cities within 24 countries.

Participants:

Aggregated city-level all-cause standardised mortality rate by gender and Western or Eastern European Region.

Results:

The proportion of specific land covers/uses within a city was related to mortality, displaying differences by macroregion and sex. The land covers/uses associated with lower SMR for both Western and Eastern European cities were those characterised by 'natural' green space, such as forests and semi-natural areas (Coef:-18.3, 95%CI -27.8 to -6.9). Dense housing was related to a higher SMR, most prominently in Western European cities (Coef:17.4, 95%CI 9.6 to 25.2).

Conclusions:

There is pressure to build on urban natural spaces, both for economic gain and because compact cities are regarded as more sustainable, yet here we offer evidence that doing so may detract from residents' health. Our study suggests that urban planners and developers need to regard retaining more wild and unstructured green space as important for healthy city systems, and that they should reconsider the push for dense and compact cities.

Keywords: urban; mortality; landscape; ecology; land use; land cover; urban landscape.

Article summary

Strengths and limitations of this study

- This was a large international study of 233 cities within 24 countries.
- We were able to utilise high quality, internationally comparable land cover/use data from the European Urban Atlas for each city to assess the configuration and quantity of these within each city.
- By including a large number of cities and countries with differing economic position, we believe our results can be generalised to similar cities globally.
- We surpass some previously research by including a very large range of land covers/uses, rather than studying the impact of just one or two.
- However we have no information on how the individuals used or were exposed to the land covers/uses that we included in our analysis.

For peer review only

For peer review only - http://bmjopen.bmj.com/site/about/guidelines.xhtml

Background

In 2014, 54% of the world's population lived in urban environments; this is set to rise to 66% by 2050[1]. It is therefore important that urban environments are designed to enable and promote good health. Urban areas are complex systems and one emergent feature is the health of their residents[2]. There are stark between-city inequalities in population health[3] and previous work on trends and inequalities between European cities in sex-specific mortality rates suggests that a city environment itself, is an important influence[4-6].

The physical extent, design and composition of cities are important components of the urban system and are associated with the health and wellbeing of residents[7-9]. The diverse development trajectories of cities means there is considerable variety in their size, configuration, and content[10]. This variety provides a rich natural laboratory to study the implications for health of their design and construction. Given that most cities are subject to some kind of planning control and vision, there is also great potential for epidemiological insights into how the urban landscape affects health to impact on the lived experiences of billions of people.

This study is about 'what is on the ground' in a city, which we refer to as land cover/uses. Land cover refers to what the surface actually is, for example forests, concrete or water. Land uses are the purposes for which humans use the land, such as; airports or roads. Urban land cover/use can influence health via multiple mechanisms including the promotion or inhibiting of healthy behaviours[11], increasing exposure to pathogens such as air pollutants and noise[12], or offering psychologically restorative spaces[13].

Evidence suggests that land cover/use exerts an independent influence on residents, over and above residents' own characteristics. For example the density of settlement and transport networks have been associated with variation in mortality risk across a number of English cities[14]. In a previous multi-city European study, we found that the presence and quantity of specific land cover/uses within a city was associated with residents' reported life satisfaction. We also found that specific land covers/uses, and a more even distribution of the land covers/uses (as opposed to dominance by one or two land covers/uses), were both associated with lower levels of socio-economic inequality in life

For peer review only

For peer review only - http://bmjopen.bmj.com/site/about/guidelines.xhtml

satisfaction within the city[15]. We hypothesised that the range and distribution of land covers/uses speaks to the range of opportunities and environments, and hence affordances[16] that the city provides – and that this might explain the associations with life satisfaction levels and its equality. However, life satisfaction is fundamentally a subjective measure of well-being, and may be particularly sensitive to the environment. Therefore in this study we focused on mortality which is, arguably, a more robust measure of population health. In doing so, we were able to include a substantially larger sample of European cities (n=233). No previous study has assessed associations between city-level land covers/uses and mortality in such a large and diverse international sample of cities.

The study aim was to determine whether the range and distribution of all, and proportions of specific, land covers/uses within European cities is associated with the city-specific mortality rate. Previous research found that a city's wider macroregion (i.e. its location in Eastern or Western Europe) was an important determinant of mortality rate, and moderator of association between environment and health[5 17], therefore this was also explored. Our specific research questions were:

i) Is the range and distribution of land covers/uses within a city associated with city-level mortality rates?

ii) Which, if any, specific land cover/uses are associated with city-level mortality rates?

iii) How do these between associations vary with sex and by macroregion?

For peer review only - http://bmjopen.bmj.com/site/about/guidelines.xhtml

For peer review only

Methods

Setting and data sources

European Urban Atlas

The 2006 Urban Atlas provided pan-European comparable land cover/use data collected and digitised from satellite data for urban areas[18]. We selected data for this period to match the time period for the available mortality data. The Atlas distinguishes 26 different land cover/use categories at a 10m^2 resolution[19]. Figure 1 provides an illustration of these data for part of one city. In this example, 20 different land uses are shown and labelled. Full land use descriptions are available in Supplementary Table 1. The land cover/use data were matched to the same city administrative boundaries as the mortality data.

Geoprocessing of Urban Atlas data

We calculated the total area (km^2) for each land cover/use by city, using an extension for ArcMap GIS called Patch Analyst[20]. This was then converted to proportion of land cover/use by city (some values equalled zero if the land cover/use was not present in a city, for example, not all cities contained an airport). Following our previous work[15], we then calculated landscape metrics. These are quantitative summaries of the range and statistical distribution of the various land covers/uses within a whole city. We calculated Shannon's diversity index (SDI) and Shannon's Evenness index (SEI)[21] to assess the variety of all land covers/uses, and their relative availability, within each city.

Gross Domestic Product (GDP)

Following Richardson *et al.* (2017)[3], the GDP per 1000 capita for the city region was used as a measure of affluence/poverty for that city. Historic GDP per country for the years 2003-2006 inclusive were extracted from Eurostat and averaged to provide a single measure for each city.

European mortality data (Standardised Mortality Ratios)

Mortality was measured using all-cause standardised mortality ratios (SMR) for males and females separately, calculated for 233 European cities by Richardson et al[3]. Values higher than 100 indicated a higher level of mortality than expected given the time period and age structure of the city population, and values lower than 100 indicated a lower level. Richardson et al's SMR data covered the time period 1999 to 2009, divided into three waves; the second of which was 2003 to 2006. This

For peer review only - http://bmjopen.bmj.com/site/about/guidelines.xhtml

time period most closely matched the date of the land cover measures from the Urban Atlas. The mortality indicator was at the city-level. No information was available about mortality rates either for socio-economic groups or spatial locations within the city. We could not, therefore, assess within-city inequalities in this study.

The SMR, land cover/use and GDP data were matched at city level. The final dataset included 233 cities (within 24 countries). The cities were allocated to either Western or Eastern-Central bloc macroregion, following Richardson et al[3], referred to hereafter as Western or Eastern. There were 85 cities (in 11 countries) classed as Eastern.

Patient and public involvement

No patients were involved in the development of the research question, design and implementation of the study, or interpretation of the results.

Statistical Analysis

First, linear regression models assessed associations between the landscape metrics and city SMR data, adjusting for city GDP. Next, models for specific land cover/uses were built. Preliminary analyses suggested that associations between the specific land cover/use measures and SMR were not linear, so the former were categorised. To do this, the proportion of the total area covered by each land measure was calculated within each city, and then classified into quintiles. This process was carried out separately for each macroregion. These quintiles were then fit as factors, along with city GDP, in a linear regression, while also allowing for clustering within country. Each land measure was assessed separately using Wald statistics and subjected to post-hoc pairwise comparisons using Sidak's correction for multiple testing across all terms[22]. All analyses were run separately by sex and macroregion. Stata(SE,14)[23] and R(v3.6.0)[24] were used for analysis, with significance levels set at 5%.

Results

For the models looking at specific land covers/uses we present our results in three ways. First, we highlight the land covers/uses for which we found strongest evidence of association with either lower

For peer review only - http://bmjopen.bmj.com/site/about/guidelines.xhtml

or higher mortality rates (Table 1). This selection was systematic, based on Wald test values and on both size and apparent ‘dose response’ of associations between quintiles of land cover/use and mortality. Table 1 crudely ‘ranks’ these selected land covers/uses according to the extent to which they were associated with mortality in both macroregions, and both sexes (which we call ‘universal’). A downward pointing arrow denotes that more of that land cover/use was associated with lower morality, and an upward arrow that it was associated with higher mortality. Second, we present regression coefficients for these selected land cover/uses (Table 2). Supplementary tables 2 (Western Europe) and 3 (Eastern Europe) provide the full set of regression coefficients for *all* land cover/uses, together with Wald statistics. Third, we graph marginal means from the adjusted regression models for associations identified in Table 1 as being either ***universal*** or ***partially universal*** (Figures 2 and 3). These figures convert the associations to estimated SMRs, perhaps a more meaningful expression of the results. The reader is invited to pay attention to the position on the Y axis, and slope, of the lines in the figures. We found no meaningful associations between SMRs and the landscape metrics and therefore do not report those results.

For peer review only - http://bmjopen.bmj.com/site/about/guidelines.xhtml

Table 1: Land cover/uses with strongest and most consistent evidence of association with city SMRs

**Universal (Both regions and sexes)**			
**West (M&F)**	***Trend***	**East (M&F)**	***Trend***
Agricultural, semi-natural areas, wetlands	↓ *SMR*	Agricultural, semi-natural areas, wetlands	↓ *SMR*
**Partially universal (across either both regions and/or sexes)**			
**West F only**	***Trend***	**East (M&F)**	***Trend***
Green urban areas	↑ *SMR*	Green urban areas	↑ *SMR*
**West (M&F)**	***Trend***	**East F only**	***Trend***
Industrial, commercial, public, military	↑ *SMR*	Industrial, commercial, public, military	↑ *SMR*
**Regional (Both sexes)**			
**West (M&F)**	***Trend***	**East (M&F)**	***Trend***
Discontinuous low density urban fabric*	↑ *SMR*		
Residential*	↑ *SMR*		
		Sports and leisure facilities	↓ *SMR*
		Land without current use	↑ *SMR*
**Sex (both regions)**			
**Males**	***Trend***	**Females (E & W)**	***Trend***
		Forests	↓ *SMR*
**Regions & opposite sex**			
**West M**	***Trend***	**East F**	***Trend***
Isolated structures	↓ *SMR*	Isolated structures	↓ *SMR*

* Most evident in cities with the highest proportion of this land cover/use (quintile 5)

view only

For peer review only - http://bmjopen.bmj.com/site/about/guidelines.xhtml

Table 2: Regression coefficients of land cover/uses found to contribute to a model for a particular macroregion/sex based on Wald test measure.

Quintiles (proportion of land-use)	West								East							
	Males				Females				Males				Females			
	Coef	LL 95% CI	UL 95% CI	p	Coef	LL 95% CI	UL 95% CI	p	Coef	LL 95% CI	UL 95% CI	p	Coef	LL 95% CI	UL 95% CI	p
**Agricultural, semi-natural areas, wetlands**																
1	ref				ref				ref				ref			
2	-10.22	-17.69	-2.75	0.011	-11.66	-20.75	-2.58	0.016	-14.45	-30.34	1.44	0.07	-3.68	-12.24	4.88	0.36
3	-11.67	-17.30	-6.04	0.001	-13.03	-23.12	-2.94	0.015	-11.51	-30.34	7.32	0.203	-2.86	-10.81	5.08	0.441
4	-12.63	-19.14	-6.13	0.001	-19.71	-33.78	-5.65	0.01	-18.67	-37.03	-0.32	0.047	-7.14	-16.67	2.39	0.126
5	-13.66	-21.08	-6.23	0.002	-18.32	-29.77	-6.88	0.004	-3.89	-26.56	18.78	0.71	5.76	-8.51	20.03	0.39
	(Wald test of parameter: 0.006)				*(Wald test of parameter: 0.051)*				*(Wald test of parameter: 0.017)*				*(Wald test of parameter: 0.014)*			
**Forests**																
1	ref				ref				ref				ref			
2	-4.60	-10.36	1.16	0.11	-9.71	-18.44	-0.98	0.03	-6.93	-20.41	6.55	0.28	-13.55	-24.82	-2.28	0.02
3	-4.53	-10.66	1.60	0.13	-8.48	-18.81	1.85	0.10	-5.82	-13.75	2.11	0.13	-10.43	-16.25	-4.62	0.00
4	-9.54	-19.44	0.37	0.06	-15.13	-31.88	1.62	0.07	-0.73	-12.67	11.21	0.89	-8.27	-19.36	2.83	0.13
5	-9.03	-16.99	-1.08	0.03	-12.59	-25.08	-0.11	0.05	-4.99	-13.08	3.10	0.20	-15.96	-24.74	-7.17	0.00
	(Wald test of parameter: 0.156)				*(Wald test of parameter: 0.005)*				*(Wald test of parameter: 0.309)*				*(Wald test of parameter: 0.010)*			
**Green urban areas**																
1	ref				ref				ref				ref			
2	1.60	-4.47	7.66	0.58	-0.02	-7.11	7.07	1.00	1.48	-6.00	8.95	0.67	4.07	-8.47	16.62	0.49
3	3.96	-2.05	9.96	0.18	1.43	-7.81	10.67	0.74	-5.99	-12.37	0.39	0.06	-3.06	-12.89	6.77	0.50
4	7.23	-0.26	14.73	0.06	9.57	1.21	17.93	0.03	-10.07	-19.96	-0.19	0.05	-9.26	-23.29	4.77	0.17
5	11.33	2.44	20.21	0.02	15.06	4.45	25.67	0.01	15.37	3.66	27.08	0.02	3.85	-8.48	16.19	0.50
	(Wald test of parameter: 0.132)				*(Wald test of parameter: 0.029)*				*(Wald test of parameter: 0.001)*				*(Wald test of parameter: 0.004)*			
**Industrial, commercial, public, military**																
1	ref				ref				ref				ref			
2	2.02	-2.51	6.55	0.35	-0.23	-6.54	6.08	0.94	-12.53	-29.98	4.91	0.14	-0.92	-18.26	16.43	0.91
3	1.34	-5.24	7.93	0.67	1.75	-6.93	10.42	0.67	-1.70	-16.73	13.32	0.81	4.72	-9.75	19.20	0.48
4	3.24	-2.16	8.65	0.22	6.76	2.31	11.21	0.01	-7.70	-20.69	5.30	0.22	-1.34	-12.77	10.08	0.80
5	10.64	3.79	17.50	0.01	11.02	0.93	21.11	0.04	-2.81	-11.94	6.31	0.51	6.09	-0.19	12.37	0.06

For peer review only - http://bmjopen.bmj.com/site/about/guidelines.xhtml

	(Wald test of parameter: 0.049)				*(Wald test of parameter: 0.023)*				*(Wald test of parameter: 0.491)*				*(Wald test of parameter: 0.033)*			
**Isolated structures**																
1		ref				ref				ref				ref		
2	-5.04	-12.40	2.32	0.16	-9.40	-19.77	0.97	0.07	-5.55	-19.15	8.04	0.38	-7.79	-18.77	3.19	0.15
					-											
3	-6.42	-10.46	-2.39	0.00	10.60	-18.98	-2.23	0.02	-2.28	-15.09	10.53	0.70	-2.19	-10.70	6.31	0.58
4	-7.94	-14.52	-1.35	0.02	-7.23	-14.35	-0.12	0.05	-5.12	-16.15	5.92	0.33	-10.66	-17.95	-3.37	0.01
5	-7.87	-15.28	-0.46	0.04	-8.46	-18.68	1.77	0.10	-6.14	-19.55	7.26	0.33	-12.35	-16.73	-7.98	<0.001
	(Wald test of parameter: 0.029)				*(Wald test of parameter: 0.145)*				*(Wald test of parameter: 0.814)*				*(Wald test of parameter: 0.001)*			
**Residential proportion**																
1		ref				ref				ref				ref		
2	1.13	-5.96	8.22	0.74	0.82	-13.05	14.68	0.90	-0.70	-16.68	15.27	15.27	-6.19	-18.52	6.15	0.29
3	2.77	-2.63	8.17	0.29	0.23	-9.54	10.00	0.96	-4.58	-16.14	6.97	6.97	-2.68	-14.96	9.60	0.64
4	3.87	-0.53	8.26	0.08	3.86	-0.96	8.68	0.11	-2.50	-13.04	8.04	8.04	-5.32	-14.18	3.54	0.21
5	13.19	7.88	18.49	<0.001	17.39	9.61	25.18	<0.001	3.56	-13.90	21.02	21.02	-0.70	-12.82	11.42	0.90
	(Wald test of parameter: <0.001)				*(Wald test of parameter: 0.004)*				*(Wald test of parameter: 0.469)*				*(Wald test of parameter: 0.155)*			
**Land without current use**																
1 (Least)		ref				ref				ref				ref		
2	0.65	-4.57	5.88	0.79	-4.16	-14.34	6.03	0.39	0.14	-6.55	6.84	0.96	-2.02	-7.45	3.41	0.43
3	1.09	-4.36	6.55	0.67	-4.20	-12.08	3.69	0.27	8.29	-2.13	18.71	0.11	11.30	3.43	19.18	0.01
4	0.98	-3.80	5.77	0.67	-1.62	-5.91	2.67	0.43	14.75	5.70	23.81	0.01	12.81	6.49	19.14	0.00
5 (Most)	10.18	0.07	20.30	0.05	4.58	-6.12	15.28	0.37	13.67	1.05	26.29	0.04	9.30	3.09	15.51	0.01
	(Wald test of parameter: 0.249)				*(Wald test of parameter: 0.316)*				*(Wald test of parameter: <0.001)*				*(Wald test of parameter: <0.001)*			
**Discontinuous low density urban fabric**																
1 (Least)		ref				ref				ref				ref		
2	0.46	-6.48	7.40	0.89	-3.85	-13.57	5.87	0.41	0.67	-14.40	15.73	0.92	0.19	-18.09	18.47	0.98
3	-0.40	-6.95	6.16	0.90	-5.65	-17.07	5.76	0.30	2.83	-9.82	15.48	0.63	1.37	-12.03	14.77	0.82
4	-0.58	-12.46	11.31	0.92	-6.61	-27.00	13.79	0.50	5.38	-8.08	18.84	0.39	0.88	-17.35	19.10	0.92
5 (Most)	7.98	2.22	13.75	0.01	7.92	-1.62	17.46	0.10	1.47	-13.93	16.87	0.84	-3.82	-24.54	16.89	0.69
	(Wald test of parameter: 0.005)				*(Wald test of parameter: 0.027)*				*(Wald test of parameter: 0.822)*				*(Wald test of parameter: 0.408)*			
**Sports and leisure facilities**																
1 (Least)		ref				ref				ref				ref		
2	1.68	-6.25	9.61	0.65	-4.11	-20.19	11.97	0.59	-7.42	0.17	-18.66	3.82	-7.20	0.13	-16.97	2.58
												-				
3	2.48	-3.15	8.12	0.36	0.01	-10.95	10.97	1.00	-18.90	0.00	-27.75	10.06	-15.15	0.04	-29.78	-0.53
4	4.60	-0.11	9.31	0.06	9.67	1.18	18.16	0.03	-13.80	0.02	-25.27	-2.33	-13.98	0.06	-28.92	0.97
5 (Most)	10.39	1.82	18.95	0.02	14.12	2.88	25.35	0.02	-19.87	0.00	-31.05	-8.68	-15.16	0.06	-31.38	1.07
	(Wald test of parameter: 0.179)				*(Wald test of parameter: 0.086)*				*(Wald test of parameter: 0.002)*				*(Wald test of parameter: 0.259)*			

For peer review only - http://bmjopen.bmj.com/site/about/guidelines.xhtml

Land cover/uses associated with lower mortality

In general, cities with a higher proportion of ***agricultural, semi-natural areas and wetlands*** enjoyed lower mortality rates (Figure 2). In Eastern cities this advantage weakened in cities with the *highest* proportion of this land-use (Figure 2, Table 2). Figure 2 shows a somewhat large difference in SMR between both sexes for Western cities in quintile 1 and quintile 5. A higher proportion of ***forests*** within a city was also associated with lower SMR for females in both Eastern and Western European cities, but not males (Table 2). In Western cities, the proportion of ***isolated structures*** *(single dwellings surrounded on all sides with natural green land)* was associated with lower SMRs for males, but in Eastern cities this protective association was present for females only (Table 2). For Eastern European cities only, higher proportion of land containing ***sports and leisure facilities*** was associated with lower SMRs, though only significantly for men (Table 2).

Land cover/uses associated with higher mortality

The two land cover/uses most consistently associated with higher mortality were **industrial, commercial, public and military**, and **green urban areas** (figure 3). In Western European cities higher proportions of the former were associated with higher SMRs, with a sharpening of association in cities containing the most (quintile 5). The association was not consistent or significant for men living in Eastern cities but was for women, though the trend fluctuated and only cities with greatest proportions of this land cover/use were associated with a higher SMR. A positive association between **green urban areas** and SMR was seen for Western women, and both sexes in Eastern cities (Table 2, Figure 3). The trend in Eastern cities suggested a threshold effect, with modest reductions in SMR as proportions increase from quintiles 1 to 4, and then a sudden and steep adverse association at quintile 5.

More derelict land (***land without current use***) was associated with higher SMRs in the Eastern cities, with the greatest adverse association quintiles 4 and 5. Western European cities with higher proportions of land classified as ***residential*** and ***discontinuous low density urban fabric*** had higher SMRs, particularly for cities in quintile 5 (Table 2).

For peer review only - http://bmjopen.bmj.com/site/about/guidelines.xhtml

Discussion

This is the first pan-European study to examine whether, and which, land cover/uses within a whole city are associated with mortality rates. By integrating health and environmental data from a large number of cities across Western and Eastern Europe we found that: the proportion of some specific land covers/uses within a city was related to mortality; most land covers/uses were not clearly associated with mortality and; there were differences by macroregion and sex in association. We found no evidence that the overall distribution and balance of multiple land cover/uses, measured by landscape metrics, was related to mortality rates.

Overall, we observed that higher proportions of natural spaces and less dense or non-residential land cover/use was associated with lower mortality. For example, greater proportions of lower density settlement (such as **isolated structures**) showed some association with lower SMR, whilst SMRs were higher for Western European cities with a greater residential proportion and low density discontinuous cover (which is principally housing). Specifically, our most consistent finding was that presence of more 'relatively wild' green spaces, such as **agricultural**, **semi-natural areas and wetlands** and **forests** was associated with lower SMRs, an association observed across sexes and macroregions and relatively strongly. The health benefits of contact with nature are very well established from both experimental and observational studies[25]. These kinds of spaces may be particularly restorative or conducive of leisure and recreation. Studies have shown the protective effects of natural environments and association with reduced risk of mortality. For example two large Canadian studies examined green space using normalized difference vegetation index (NDVI) around residents' home and showed a higher NDVI index was associated with lower mortality rates[26 27]. Our results for the more natural spaces are in stark contrast to those for **green urban areas**, the proportion of which was relatively consistently associated with *higher* mortality. At first, this seems like a directly contradictory finding, but it is important to be clear what kinds of spaces are classified as '**green urban areas'** in the Urban Atlas. In this land use classification, they are relatively small and manicured 'in-fill' green spaces within residential and commercial developments. Their association with greater mortality is not unprecedented in the literature. Previous work found that the

For peer review only - http://bmjopen.bmj.com/site/about/guidelines.xhtml

total amount of green space within American cities was associated with higher mortality rates, suggesting that the indicator was capturing sprawling cities, however this study was unable to distinguish between the green space type[28]. In light of our findings, the American study may have been capturing the development of semi-natural areas into 'in-fill' green developments. We have previously found that greater amounts of '**green urban areas**' in European cities were associated with reduced reported life satisfaction for its city residents[15]. The salutogenic effects of nature may be maximised by more natural areas and / or the 'pockets of green' in and among urban developments may be insufficient to offset the adversity of living in dense residential and commercial environments.

There were some differences between region and sex in the direction of associations. We found that less desirable land types/uses within cities, such as **land without use**, were associated with higher SMRs for those living in Eastern European cities. Dereliction of cities has been linked to decreasing employment rates due to many individuals, particularly younger and educated, migrating from these areas to more prosperous cities[29] and individuals living in areas with a high proportion of brownfield land are significantly more likely to suffer from poorer health than those with a lower proportion[30]. Dereliction was a national problem in many Western cities during the mid/late 20th century as many industries declined or closed[29], but may now be less important for population health. Perhaps the approaches taken by some Western governments[31 32] to tackle it and spearhead regeneration in these areas for example, have mitigated impacts. Further, the impact of **industrial, commercial, public and military** spaces within a city seemed benign for men living in Eastern European Cities but not for women, and both sexes in Western European cities. Males in eastern cities also seemed to benefit from greater proportions of **sports and leisure facilities**. The western / eastern differences may reflect levels of city and economic development not adequately captured by our control for GDP. Perhaps, for example, a city in the east that has substantial amounts of land dedicated to **sports and leisure facilities** hosts a population with sufficient affluence to sustain them.

Overall, our results present a challenge to healthy urban planning. Building on natural green spaces can bring money to city coffers, and help swell urban populations. Residential settlements with 'green

For peer review only - http://bmjopen.bmj.com/site/about/guidelines.xhtml

views' command a premium price. Further, a central message of contemporary urban planning is that dense and/or compact cities are 'sustainable'. Yet, the literature already hints that compact cities characterised by high density residential areas have both benefits and disadvantages for their residents. The environmental benefits, owing to the reduced carbon emissions required in intraurban transport and service, have been well described[4], in contrast, population quality of life measures often show poor outcomes[33].

Strengths and weaknesses

This was a large international study of 233 cities within 24 countries. We were able to utilise high quality, internationally comparable land cover/use data from the European Urban Atlas for each city to assess the configuration and quantity of these within each city. By including a large number of cities and countries with differing economic position, we believe our results can be generalised to similar cities globally. We were able to dissect the impact of land covers/uses both collectively (via landscape metrics) and individually. We surpass some previously research by including a very large range of land covers/uses, rather than studying the impact of just one or two[34]. We were able to match previously validated SMR data to land cover/use data for the same areas and a close time point. SMRs are robust, valid and widely used measures of population health.

However, although we described associations between land covers/uses and SMRs, we have no information on how the individuals used or were exposed to the land covers/uses that we included in our analysis, for example if they live in a part of the city without that particular land use. The development of the sample cities may also have changed considerably during individuals' life courses and the individual may not have always resided in that city, thus impacting on SMRs. The mortality indicator we used for the study was a city-level population level measure and therefore we were unable to explore inequality in mortality by socio-economic status.

Conclusion

We found that the proportion of specific land cover/uses within cities is associated with mortality rates there, but that these associations varied by macroregion and by sex. There is great pressure to build on wild green space for economic gain and sustainability, and to promote compact cities as

For peer review only - http://bmjopen.bmj.com/site/about/guidelines.xhtml

sustainable. Our study suggests that urban planners and developers need to regard retaining more wild and unstructured green space as important for healthy city systems, and that they should reconsider the push for dense and compact cities.

Contributors: JRO, NN and RM contributed to the conception and design of the study. NN performed the statistical analysis. JRO performed the geospatial analysis. RM, GM, JP & NS were involved in previous work developing the main outcome measure. JRO prepared the first draft of the manuscript, with all authors contributing to its main content and revising it with critical comments. All authors have read and approved the manuscript prior to submission, and agree to be accountable for all aspects of the work in ensuring that questions related to the accuracy or integrity of any part of the work are appropriately investigated and resolved.

Funding statement: JO, NN and RM are employed by the University of Glasgow and funded as part of the Neighbourhoods and Communities Programme (MC_UU_12017/10) (SPHSU10) at the MRC/CSO Social and Public Health Sciences Unit (SPHSU).

Competing interests: None declared.

Data sharing statement*:* Standardised Mortality Ratio data are available by emailing richard.mitchell@glasgow.ac.uk. European Urban Atlas data are freely available from the European Environment Agency (https://www.eea.europa.eu/data-and-maps/data/urban-atlas), provided by the European Commission of the European Union.

Patient consent for publication: None required.

For peer review only

References

1. United Nations. World Urbanization Prospects: The 2014 Revision, Highlights. Department of Economic and Social Affairs. *Population Division, United Nations* 2014
2. Frumkin H. Urban sprawl and public health. *Public Health Rep* 2002;117(3):201-17.
3. Richardson EA, Moon G, Pearce J, et al. Multi-scalar influences on mortality change over time in 274 European cities. *Soc Sci Med* 2017;179:45-51.
4. Jabareen YR. Sustainable urban forms: Their typologies, models, and concepts. *J Plan Educ Res* 2006;26(1):38-52.
5. Marí-Dell'Olmo M, Gotsens M, Palència L, et al. Socioeconomic inequalities in cause-specific mortality in 15 European cities. *J Epidemiol Community Health* 2015;69(5):432-41.
6. Gotsens M, Marí-Dell'Olmo M, Pérez K, et al. Socioeconomic inequalities in injury mortality in small areas of 15 European cities. *Health Place* 2013;24:165-72.
7. Ewing R, Schmid T, Killingsworth R, et al. Relationship between urban sprawl and physical activity, obesity, and morbidity. Urban Ecology: Springer 2008:567-82.
8. Sallis JF, Floyd MF, Rodríguez DA, et al. Role of built environments in physical activity, obesity, and cardiovascular disease. *Circulation* 2012;125(5):729-37.
9. Sarkar C, Webster C, Gallacher J. Healthy cities: public health through urban planning: Edward Elgar Publishing 2014.
10. Lawrence RJ. Human Ecology in the Context of Urbanisation. In: Nieuwenhuijsen M, Khreis H, eds. Integrating Human Health into Urban and Transport Planning. Cham, Switzerland: Springer 2018.
11. Giles-Corti B, Vernez-Moudon A, Reis R, et al. City planning and population health: a global challenge. *Lancet* 2016;388(10062):2912-24.
12. Dadvand P, Ostro B, Figueras F, et al. Residential proximity to major roads and term low birth weight: the roles of air pollution, heat, noise, and road-adjacent trees. *Epidemiology* 2014;25(4):518-25.
13. Hunter R, Cleland C, Cleary A, et al. Environmental, health, wellbeing, social and equity effects of urban green space interventions: A meta-narrative evidence synthesis. *Environ Int* 2019;130:104923.
14. Fecht D, Fortunato L, Morley D, et al. Associations between urban metrics and mortality rates in England. *Environ Health* 2016;15(1):S34.
15. Olsen JR, Nicholls N, Mitchell R. Are urban landscapes associated with reported life satisfaction and inequalities in life satisfaction at the city level? A cross-sectional study of 66 European Cities *Soc Sci Med* 2018
16. Lennon M, Douglas O, Scott M. Urban green space for health and well-being: developing an 'affordances' framework for planning and design. *J Urban Des* 2017;22(6):778-95.
17. Marmot M, Allen J, Bell R, et al. WHO European review of social determinants of health and the health divide. *Lancet* 2012;380(9846):1011-29.
18. Copernicus. Urban Atlas 2018 [Available from: https://land.copernicus.eu/local/urban-atlas accessed 11/01/2018 2018.
19. European Commission. Mapping guide for a European urban atlas: European Commission; 2012 [Available from: http://ec.europa.eu/regional_policy/sources/tender/pdf/2012066/annexe2.pdf accessed 11/01/2018 2018.
20. Patch Analyst and Patch Grid [program]. Centre for Northern Forest Ecosystem Research, Thunder Bay, Ontario.: Ontario Ministry of Natural Resources, 2012.
21. Rempel R, Carr A, Elkie P. Patch analyst for ArcGIS®. *Centre for Northern Forest Ecosystem Research, Ontario Ministry of Natural Resources Lakehead University* 2008
22. Abdi H. Bonferroni and Šidák corrections for multiple comparisons In: Salkind NJ, ed. Encyclopedia of measurement and statistics. Thousand Oaks (CA):: Sage 2007.
23. Stata Statistical Software: Release 14. [program]. College Station, TX: StataCorp LP: StataCorp., 2015.
24. R: A language and environment for statistical computing. [program]: R Foundation for Statistical Computing, 2018.
25. Hartig T, Mitchell R, De Vries S, et al. Nature and health. *Annu Rev Public Health* 2014;35:207-28.

For peer review only - http://bmjopen.bmj.com/site/about/guidelines.xhtml

26. Crouse DL, Pinault L, Balram A, et al. Urban greenness and mortality in Canada's largest cities: a national cohort study. *Lancet Planet Health* 2017;1(7):e289-e97.
27. Villeneuve PJ, Jerrett M, Su JG, et al. A cohort study relating urban green space with mortality in Ontario, Canada. *Environ Res* 2012;115:51-58.
28. Richardson EA, Mitchell R, Hartig T, et al. Green cities and health: a question of scale? *J Epidemiol Community Health* 2012;66(2):160-65.
29. Ward W. Environmental Improvements in Relation to Derelict Land. *J R Soc Health* 1970;90(4):208-11.
30. Bambra C, Robertson S, Kasim A, et al. Healthy land? An examination of the area-level association between brownfield land and morbidity and mortality in England. *Environ Plan A* 2014;46(2):433-54.
31. Northern Ireland Executive. Urban Development Grant Scheme 2019 [Available from: https://www.communities-ni.gov.uk/articles/urban-development-grant-scheme accessed 29.04.2019 2019.
32. Scottish Goverment. Capital investment for regeneration Edinburgh, Scotland: Scottish Government; 2019 [Available from: https://www.gov.scot/policies/regeneration/capital-investment/ accessed 29.04.2019 2019.
33. Neuman M. The compact city fallacy. *J Plan Educ Res* 2005;25(1):11-26.
34. Litman T, Steele R. Land use impacts on transport: How land use factors affect travel behavior: Victoria Transport Policy Institute Victoria, BC., Canada, 2012.

For peer review only - http://bmjopen.bmj.com/site/about/guidelines.xhtml

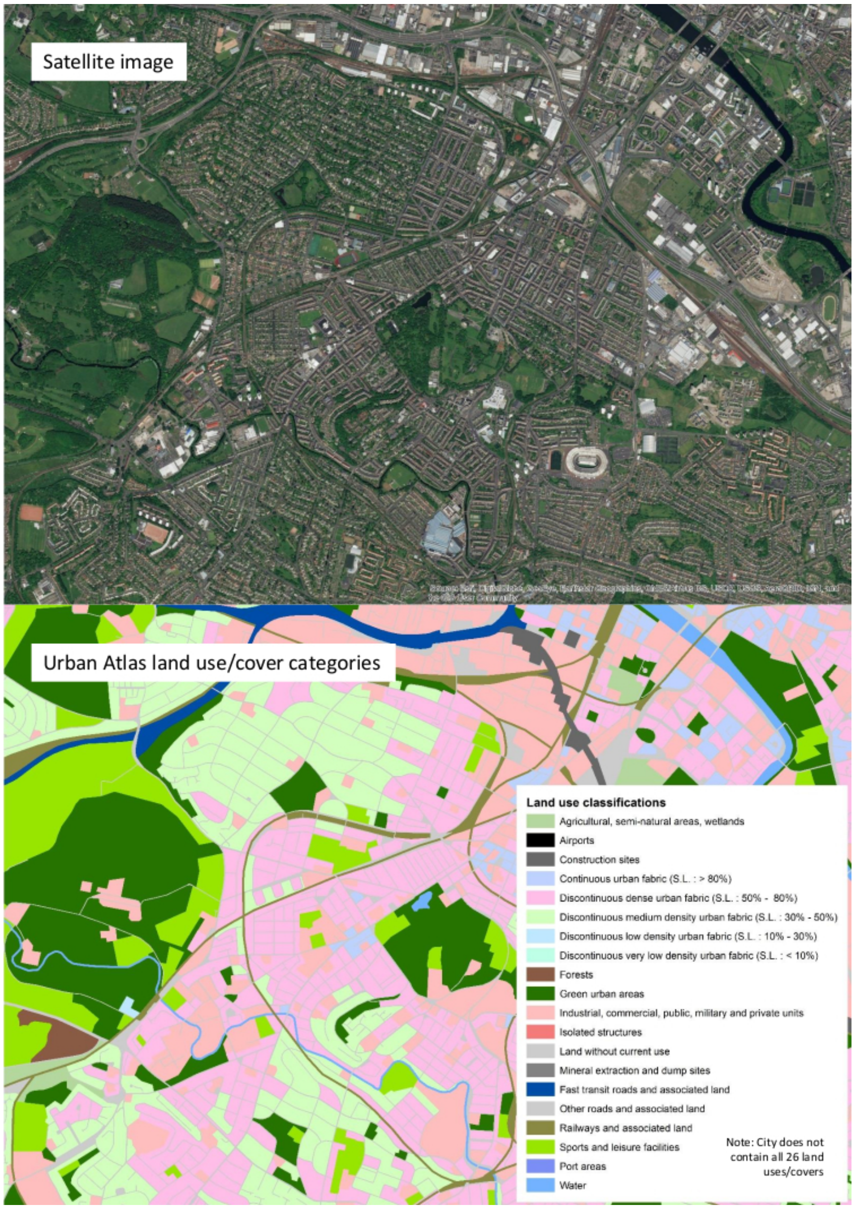

Figure 1: Urban Atlas land use/cover categories and satellite base map.

190x269mm (100 x 100 DPI)

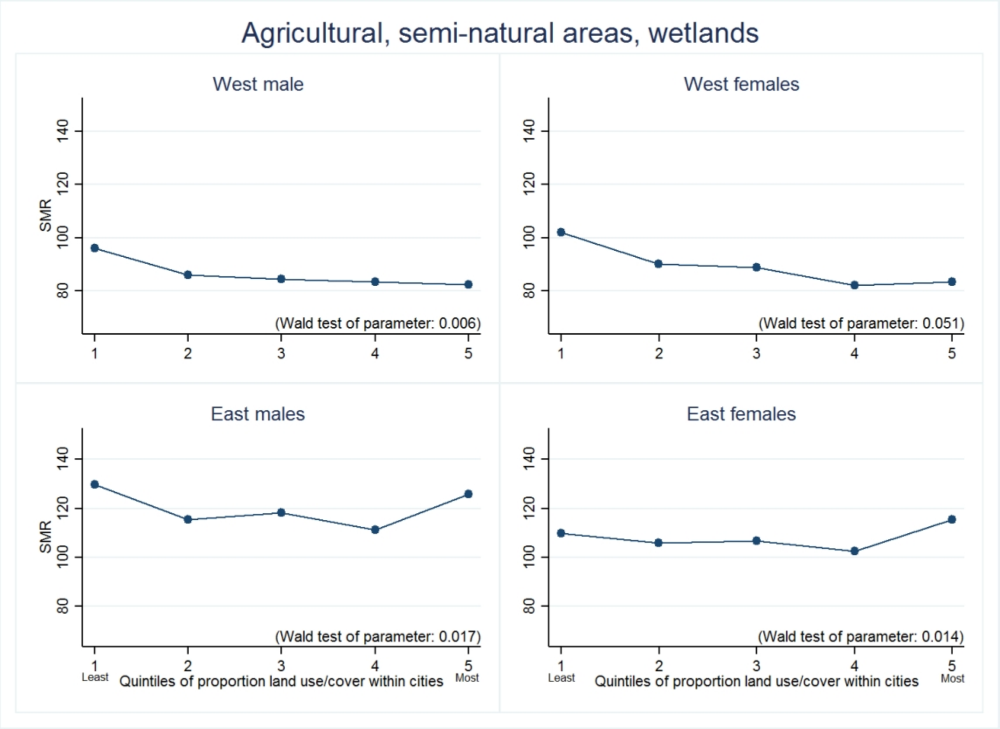

Figure 2: Estimated SMRs for cities with varying quintiles of Agricultural, semi-natural areas, wetlands (quintile 1 = lowest proportion)

385x280mm (96 x 96 DPI)

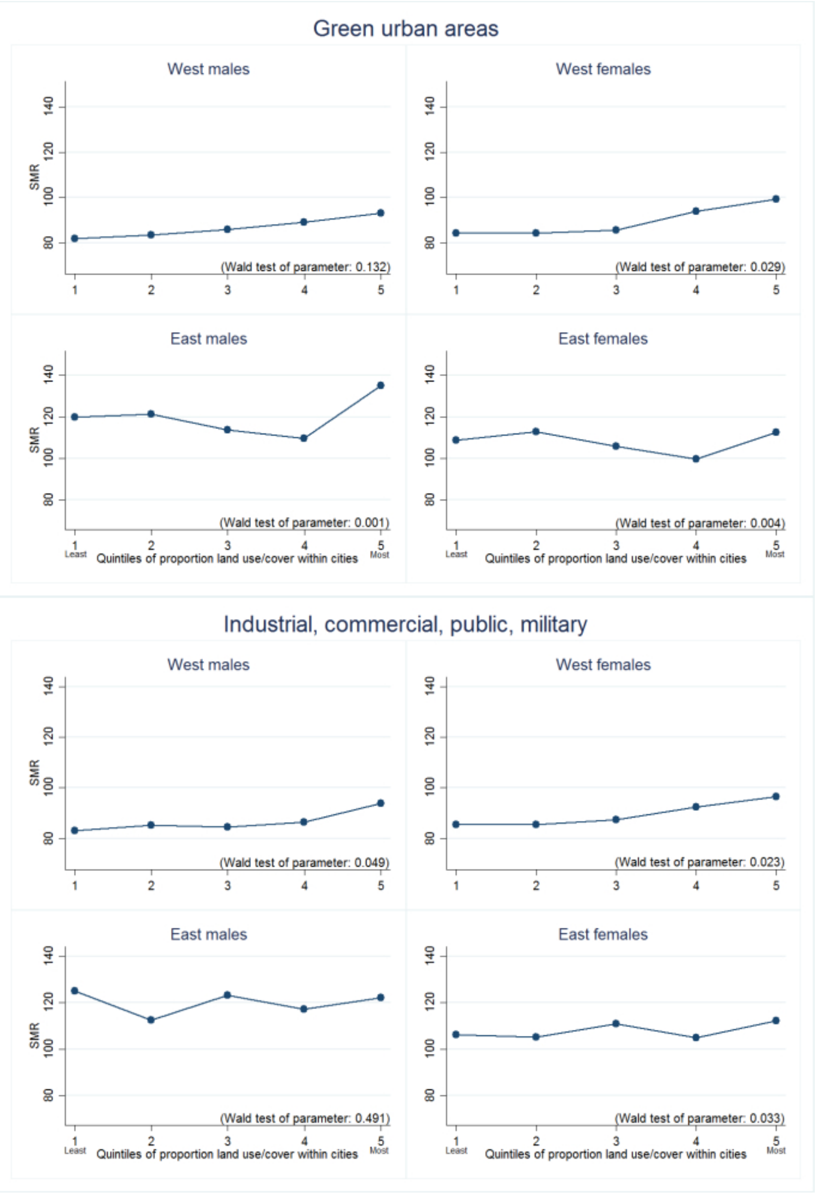

Figure 3: Comparisons between SMR and quintiles land cover/uses for Green urban areas and Industrial, commercial, public, military (partially universal importance by macroregion and/or sex).

173x254mm (100 x 100 DPI)

Which urban land covers/uses are associated with residents' mortality? A cross-sectional, ecological, pan-European study of 233 cities.

Supplementary Table 1: European Urban Atlas land-use definitions.

**1. Artificial surfaces**		Buildings, roads, all constructions of infrastructure and other artificially sealed or paved areas.
**1.1**	**Urban fabric**	Built-up areas and their associated land, such as gardens, parks, planted areas and non-surfaced public areas and the infrastructure, if these areas are not suitable to be mapped separately with regard to the minimum mapping unit size.
1.1.1	Continuous urban fabric	Predominant residential use: areas with a high degree of soil sealing, independent of their housing scheme (single family houses or high rise dwellings, city centre or suburb).
1.1.2	Discontinuous urban fabric	Predominant residential usage. Contains more than 20% non-sealed areas, independent of their housing scheme (single family houses or high-rise dwellings, city centre or suburb). The non-sealed areas might be private gardens or common green areas.
1.1.2.1	Discontinuous dense urban fabric	Residential buildings, roads and other artificially surfaced areas (Average degree of soil sealing: > 50 - 80%).
1.1.2.2	Discontinuous medium density urban fabric	Residential buildings, roads and other artificially surfaced areas. The vegetated areas are predominant, but the land is not dedicated to forestry or agriculture (Average degree of soil sealing: > 30 - 50%).
1.1.2.3	Discontinuous low density urban fabric	Residential buildings, roads and other artificially surfaced areas. The vegetated areas are predominant, but the land is not dedicated to forestry or agriculture (Average degree of soil sealing: 10 - 30%).
1.1.2.4	Discontinuous very low density urban fabric	Residential buildings, roads and other artificially surfaced areas. The vegetated areas are predominant, but the land is not dedicated to forestry or agriculture. Example: exclusive residential areas with large gardens (Average degree of soil sealing: <10 %).
1.1.3	Isolated structures	Isolated artificial structures with a residential component, such as (small) individual farm houses and related buildings. The mapping unit will never be surrounded by any urban class other than transportation network.
**1.2**	**Industrial, commercial, public, military, private and transport units**	At least 30% of the ground is covered by artificial surfaces. More than 50% of those artificial surfaces are occupied by buildings and / or artificial structures with non-residential use, i.e. industrial, commercial or transport related uses are dominant.
1.2.1	Industrial, commercial, public, military and private units	Artificial structures (e.g. buildings) or artificial surfaces (e.g. concrete, asphalt, tar, macadam, tarmac or otherwise stabilised surface, e.g. compacted soil, devoid of vegetation), occupy most of the surface.
1.2.2	Road and rail network and associated land	The road and railway network (COTS navigation data) is ingested into the classification database according to the

For peer review only - http://bmjopen.bmj.com/site/about/guidelines.xhtml

		method given in the Annex within the link noted at the end of this table.
1.2.2.1	Fast transit roads and associated land	Roads defined as "motorways" in the COTS navigation data, and motorway rest and service areas and parking areas, only accessible from the motorways.
1.2.2.2	Other roads and associated land	Roads, crossings, intersections and parking areas, including roundabouts and sealed areas with "road surface".
1.2.2.3	Railways and associated land	Railway facilities including stations, cargo stations and service areas.
1.2.3	Port areas	Administrative area of inland harbours and sea ports. Infrastructure of port areas, including quays, dockyards, transport and storage areas and associated areas.
1.2.4	Airports	Administrative area of airports, mostly fenced. Included are all airport installations: runways, buildings and associated land.
**1.3**	**Mine, dump and construction sites**	Open pit extraction sites (sand, quarries) including water surface. Public, industrial or mine dump sites, raw or liquid wastes, legal or illegal, their protecting dikes and / or vegetation belts and associated land such as service areas.
1.3.3	Construction sites	Spaces under construction or development, soil or bedrock excavations for construction purposes or other earthworks visible in the image.
1.3.4	Land without current use	Areas in the vicinity of artificial surfaces still waiting to be used or re-used.
**1.4**	**Artificial non-agricultural vegetated areas**	Vegetation planted and regularly worked by humans; strongly human-influenced. Sporting facilities as functional units independent of being non-sealed, sealed or built-up.
1.4.1	Green urban areas	Public green areas for predominantly recreational use such as gardens, zoos, parks, castle parks. Suburban natural areas that have become and are managed as urban parks. Forests or green areas extending from the surroundings into urban areas are mapped as green urban areas when at least two sides are bordered by urban areas and structures, and traces of recreational use are visible.
1.4.2	Sports and leisure facilities	All sports and leisure facilities including associated land, whether public or commercially managed: e.g. Theresienwiese (Munich), public arenas for any kind of sports including associated green areas, parking places.
**2. Agricultural + semi-natural + wetland areas**		
2.1	Arable land	Fields under rotation system. Can be non-irrigated or permanently irrigated. Also includes rice fields; Fields laid in fallow are included.
2.2	Permanent crops	Fruit orchards, scattered fruit trees with pasture; Vineyards and their nurseries; Roses; Olive groves; Berries and hop plantations.
2.3	Pasture & natural grassland	Grassland; Pasture and meadow under agricultural use, grazed or mechanically harvested.
2.4	Shrubs and / or herbaceous vegetation	Vegetation cover more than 50%, ground coverage of trees with height > 5 m: < 30%, areas with minor / without

For peer review only - http://bmjopen.bmj.com/site/about/guidelines.xhtml

	including transitional woodland	artificial or agricultural influence; Sclerophyllous vegetation; Bushy sclerophyllous vegetation (e.g. maquis, garrigue); Abandoned arable land with bushes; Woodland degradation: storm, snow, insects or air pollution; Areas under power transmission lines inside forest; Fire breaks; Steep bushy slopes of eroded areas; Abandoned vineyards or orchards, arable land and pasture land under natural colonisation; Dehesas with bush proliferation indicating no agricultural or farming use for a rather long time; Bushy areas along creeks.
2.5	Moors and heathland	Bushes, shrubs and herbaceous plants, dwarf forest in alpine or coastal regions (Pinus Mugo forests). Height is maximum 3 m in climax stage.
2.6	Beaches, dunes, sand	< 10% vegetation cover; Beaches, dunes and sand plains, (coastal or inland location), gravel along rivers; Seasonal rivers, if water is characteristic for a shorter part of the year (< 2 months).
2.7	Bare rocks	> 90% of the land surface of bare rocks, (i.e. < 10% vegetation); Rocks, gravel fields, landslides; Scree (fragments resulting from mechanical and chemical erosion. Weathering rocks forming heaps of coarse debris at the foot of steep slopes), cliffs, rocks.
2.8	Sparsely vegetated areas	Steppes, tundra, badlands, scattered high altitude vegetation. Bare soils inside military training areas. Vegetation cover 10 - 50%.
2.9	Burnt areas	Recently burnt forest or shrubs (but not natural grassland), still mainly black on EO data.
2.10	Snow and ice	Glacier and perpetual snow
2.11	Inland wetlands	Areas flooded or liable to flooding during a large part of the year by fresh, brackish or standing water with specific vegetation coverage made of low shrub, semi-ligneous or herbaceous species; Water fringe vegetation, reed beds of lakes, rivers and brooks. Sedge and fen-sedge beds, swamps; Peat bogs, with or without peat extracting areas; Shallow water areas covered with reed; Seasonal rivers, if water course is not visible in the EO data.
2.12	Coastal wetlands	Areas, flooded or liable to flooding during a large part of the year by brackish or saline water, susceptible to flooding by sea water. Often in the process of filling in and gradually being colonised by halophytic plants; Specific vegetation coverage made of low shrub, semi-ligneous or herbaceous species; Alluvial planes, marshes and intertidal flats; Salinas (salt production sites by evaporation).
**3. Forest (natural and plantation)**		With ground coverage of tree canopy > 30%, tree height > 5 m, including bushes and shrubs at the fringe of the forest; Included are plantations such as Populus plantations, Christmas tree plantations; Forest regeneration / re-colonisation: clear cuts, new forest plantations.

For peer review only - http://bmjopen.bmj.com/site/about/guidelines.xhtml

**4. Water**	The visible water surface area on the EO data is delineated: Sea; Lakes; Fish ponds (natural, artificial); Rivers, including channelled rivers; Canals.

Adapted from: http://ec.europa.eu/regional_policy/sources/tender/pdf/2012066/annexe2.pdf

For peer review only

Which urban land covers/uses are associated with residents' mortality? A cross-sectional, ecological, pan-European study of 233 cities.

Supplementary Table 2: Associations between land cover/use proportion and SMR for Western European cities

Quintiles (proportion of land-use)	Males				Females			
	Coef	p	LL 95% CI	UL 95% CI	Coef	p	LL 95% CI	UL 95% CI
**Agricultural, semi-natural areas, wetlands**								
1 (Least)			ref				ref	
2	-10.22	0.01	-17.69	-2.75	-11.66	0.02	-20.75	-2.58
3	-11.67	0.00	-17.30	-6.04	-13.03	0.02	-23.12	-2.94
4	-12.63	0.00	-19.14	-6.13	-19.71	0.01	-33.78	-5.65
5 (Most)	-13.66	0.00	-21.08	-6.23	-18.32	0.00	-29.77	-6.88
	(Wald test of parameter: 0.006)				*(Wald test of parameter: 0.052)*			
**Airports ***								
1 (Least)			ref				Ref	
2	-2.87	0.29	-8.50	2.76	-3.03	0.46	-11.66	5.61
3	-8.70	0.00	-13.48	-3.93	-10.73	0.02	-19.09	-2.36
4 (Most)	-2.48	0.43	-8.97	4.02	-6.72	0.21	-17.71	4.26
	(Wald test of parameter: 0.005)				*(Wald test of parameter: 0.062)*			
**Construction sites**								
1 (Least)			ref				ref	
2	-3.07	0.37	-10.22	4.07	-9.93	0.13	-23.26	3.40
3	-1.44	0.67	-8.65	5.76	-8.35	0.16	-20.32	3.61
4	-0.68	0.91	-13.98	12.61	-12.36	0.09	-27.14	2.42
5 (Most)	-1.20	0.74	-8.84	6.44	-9.11	0.20	-23.68	5.45
	(Wald test of parameter: 0.814)				*(Wald test of parammeter:0.358)*			
**Continuous urban fabric**								
1 (Least)			ref				ref	
2	1.56	0.60	-4.72	7.84	-5.62	0.10	-12.45	1.21
3	0.00	1.00	-6.03	6.03	-4.66	0.30	-13.98	4.66
4	4.24	0.12	-1.31	9.78	-0.43	0.92	-9.09	8.23
5 (Most)	3.03	0.39	-4.26	10.31	0.09	0.99	-13.58	13.77
	(Wald test of parameter: 0.554)				*(Wald test of parameter: 0.1715)*			
**Discontinuous dense urban fabric**								
1 (Least)			ref				ref	
2	-2.38	0.59	-11.58	6.817	-5.73	0.42	-20.69	9.23
3	-3.39	0.47	-13.27	6.49	-4.45	0.63	-23.80	14.90
4	2.11	0.51	-4.66	8.87	2.28	0.73	-11.88	16.45
5 (Most)	7.36	0.15	-3.02	17.74	9.55	0.26	-7.89	26.99
	(Wald test of parameter: 0.051)				*(Wald test of parameter: 0.093)*			
**Discontinuous medium density urban fabric**								
1 (Least)			ref				ref	

For peer review only

For peer review only - http://bmjopen.bmj.com/site/about/guidelines.xhtml

2	-0.27	0.95	-8.74	8.19	-7.70	0.19	-19.80	4.39
3	-3.20	0.29	-9.40	2.99	-5.60	0.19	-14.34	3.14
4	-4.87	0.10	-10.89	1.16	-11.30	0.04	-21.67	-0.93
5 (Most)	-3.54	0.38	-12.00	4.91	-13.12	0.01	-22.56	-3.67
	(Wald test of parameter: 0.456)				*(Wald test of parameter: 0.097)*			
**Discontinuous low density urban fabric**								
1 (Least)			ref				ref	
2	0.46	0.89	-6.48	7.40	-3.85	0.41	-13.57	5.87
3	-0.40	0.90	-6.95	6.16	-5.65	0.30	-17.07	5.76
4	-0.58	0.92	-12.46	11.31	-6.61	0.50	-27.00	13.79
5 (Most)	7.98	0.01	2.22	13.75	7.92	0.10	-1.62	17.46
	(Wald test of parameter: 0.005)				*(Wald test of parameter: 0.027)*			
**Discontinuous very low density urban fabric**								
1 (Least)			ref				ref	
2	1.10	0.83	-9.74	11.93	-1.87	0.78	-16.24	12.50
3	-5.35	0.25	-14.90	4.212	-13.21	0.10	-29.21	2.79
4	-5.37	0.16	-13.18	2.45	-13.85	0.01	-24.06	-3.65
5 (Most)	-5.75	0.08	-12.28	0.77	-12.42	0.03	-22.99	-1.85
	(Wald test of parameter: 0.313)				*(Wald test of parameter: 0.034)*			
**Residential proportion**								
1 (Least)			ref				ref	
2	1.13	0.74	-5.96	8.22	0.82	0.90	-13.05	14.68
3	2.77	0.29	-2.63	8.17	0.23	0.96	-9.54	10.00
4	3.87	0.08	-0.53	8.26	3.86	0.11	-0.96	8.68
5 (Most)	13.19	0.00	7.88	18.49	17.39	0.00	9.61	25.18
	(Wald test of parameter: <0.001)				*(Wald test of parameter: 0.004)*			
**Fast transit roads and associated land**								
1 (Least)			ref				ref	
2	-5.44	0.24	-14.87	4.00	-14.64	0.03	-27.59	-1.69
3	-3.46	0.25	-9.66	2.74	-10.80	0.03	-20.54	-1.07
4	-3.61	0.41	-12.81	5.59	-15.33	0.02	-28.11	-2.55
5 (Most)	0.62	0.85	-6.44	7.68	-6.77	0.16	-16.47	2.93
	(Wald test of parameter: 0.722)				*(Wald test of parameter: 0.126)*			
**Forests**								
1 (Least)			ref				ref	
2	-4.60	0.11	-10.36	1.158271	-9.71	0.03	-18.44	-0.97842
3	-4.53	0.13	-10.66	1.600225	-8.48	0.10	-18.81	1.854112
4	-9.54	0.06	-19.44	0.372522	-15.13	0.07	-31.88	1.617823
5 (Most)	-9.03	0.03	-16.99	-1.07548	-12.59	0.05	-25.08	-0.10597
	(Wald test of parameter: 0.1562)				*(Wald test of parameter: 0.0048)*			
**Green urban areas**								
1 (Least)			ref				ref	
2	1.60	0.58	-4.47	7.66	-0.02	1.00	-7.11	7.07
3	3.96	0.18	-2.05	9.96	1.43	0.74	-7.81	10.67
4	7.23	0.06	-0.26	14.73	9.57	0.03	1.21	17.93
5 (Most)	11.33	0.02	2.44	20.21	15.06	0.01	4.45	25.67

For peer review only

	(Wald test of parameter: 0.132)				*(Wald test of parameter: 0.027)*			
**Industrial, commercial, public, military**								
1 (Least)			ref				ref	
2	2.02	0.35	-2.51	6.55	-0.23	0.94	-6.54	6.08
3	1.34	0.67	-5.24	7.93	1.75	0.67	-6.93	10.42
4	3.24	0.22	-2.16	8.65	6.76	0.01	2.31	11.21
5 (Most)	10.64	0.01	3.79	17.50	11.02	0.04	0.93	21.11
	(Wald test of parameter: 0.049)				*(Wald test of parameter: 0.023)*			
**Isolated structures**								
1 (Least)			ref				ref	
2	-5.04	0.16	-12.40	2.32	-9.40	0.07	-19.77	0.97
3	-6.42	0.00	-10.46	-2.39	-10.60	0.02	-18.98	-2.23
4	-7.94	0.02	-14.52	-1.35	-7.23	0.05	-14.35	-0.12
5 (Most)	-7.87	0.04	-15.28	-0.46	-8.46	0.10	-18.68	1.77
	(Wald test of parameter: 0.029)				*(Wald test of parameter: 0.145)*			
**Land without current use**								
1 (Least)			ref				ref	
2	0.65	0.79	-4.57	5.88	-4.16	0.39	-14.34	6.07
3	1.09	0.67	-4.36	6.55	-4.20	0.27	-12.08	3.69
4	0.98	0.67	-3.80	5.77	-1.62	0.43	-5.91	2.67
5 (Most)	10.18	0.05	0.07	20.30	4.58	0.37	-6.12	15.28
	(Wald test of parameter: 0.249)				*(Wald test of parameter: 0.316)*			
**Mineral extraction and dump sites**								
1 (Least)			ref				ref	
2	-3.71	0.22	-9.90	2.47	-6.17	0.11	-13.97	1.63
3	-5.83	0.12	-13.36	1.70	-8.17	0.23	-22.21	5.88
4	-2.74	0.42	-9.85	4.37	-5.45	0.10	-12.10	1.19
5 (Most)	-2.92	0.27	-8.40	2.56	-7.98	0.01	-13.45	-2.52
	(Wald test of parameter: 0.081)				*(Wald test of parameter: 0.061)*			
**Other roads and associated land**								
1 (Least)			ref				ref	
2	-0.95	0.71	-6.38	4.494284	-6.44	0.14	-15.22	2.330186
3	0.64	0.80	-4.66	5.935122	0.46	0.89	-6.43	7.363465
4	2.15	0.39	-3.02	7.322068	4.22	0.15	-1.79	10.23612
5 (Most)	9.49	0.02	1.55	17.42118	6.69	0.17	-3.24	16.61991
	(Wald test of parameter: 0.1889)				*(Wald test of parameter: 0.2091)*			
**Railways and associated land**								
1 (Least)			ref				ref	
2	1.12	0.81	-8.66	10.90	-5.72	0.42	-20.40	8.95
3	2.41	0.31	-2.51	7.31	-2.99	0.66	-17.47	11.49
4	5.82	0.06	-0.21	11.86	-2.74	0.44	-10.10	4.62
5 (Most)	1.81	0.56	-4.71	8.32	-7.73	0.10	-17.10	1.64
	(Wald test of parameter: 0.190)				*(Wald test of parameter: 0.2796)*			
**Sports and leisure facilities**								
1 (Least)			ref				ref	
2	1.68	0.65	-6.25	9.61	-4.11	0.59	-20.19	11.97

For peer review only - http://bmjopen.bmj.com/site/about/guidelines.xhtml

3	2.48	0.36	-3.15	8.12	0.01	1.00	-10.95	10.97
4	4.60	0.06	-0.11	9.31	9.67	0.03	1.18	18.16
5 (Most)	10.39	0.02	1.82	18.95	14.12	0.02	2.88	25.35
	(Wald test of parameter: 0.179)				*(Wald test of parameter: 0.086)*			
**Water bodies**								
1 (Least)			ref				ref	
2	5.33	0.18	-2.70	13.36	6.06	0.45	-10.56	22.69
3	-1.35	0.70	-8.79	6.08	2.29	0.68	-9.55	14.12
4	0.31	0.91	-5.31	5.93	4.27	0.34	-4.96	13.51
5 (Most)	-1.60	0.53	-6.98	3.79	2.32	0.66	-8.86	13.51
	(Wald test of parameter: 0.317)				*(Wald test of parameter: 0.467)*			

* Due to low proportion of land area classed as airports within cities, the first two quintiles combined within the model, essentially representing less than ~0.3% of the total area.

Supplementary Table 3: Associations between land cover/use proportion and SMR for Eastern European cities

Quintiles (proportion of land-use)	Males				Females			
	Coef	p	LL 95% CI	UL 95% CI	Coef	p	LL 95% CI	UL 95% CI
**Agricultural, semi-natural areas, wetlands**								
1 (Least)			ref				ref	
2	-14.45	0.07	-30.34	1.44	-3.68	0.36	-12.24	4.88
3	-11.51	0.20	-30.34	7.32	-2.86	0.44	-10.81	5.08
4	-18.67	0.05	-37.03	-0.32	-7.14	0.13	-16.67	2.39
5 (Most)	-3.89	0.71	-26.56	18.78	5.76	0.39	-8.51	20.03
	(Wald test of parameter: 0.017)				*(Wald test of parameter: 0.014)*			
**Airports ***								
1 (Least)			ref				ref	
2	7.86	0.19	-4.52	20.25	8.08	0.05	0.24	15.92
3 (Most)	15.35	0.02	2.44	28.27	8.64	0.01	2.70	14.58
	(Wald test of parameter: 0.047)				*(Wald test of parameter: 0.008)*			
**Construction sites**								
1 (Least)			ref				ref	
2	0.44	0.94	-11.40	12.27177	-3.87	0.19	-9.93	2.201498
3	1.91	0.66	-7.51	11.33426	0.88	0.80	-6.53	8.280001
4	-4.12	0.52	-17.96	9.71732	-5.57	0.26	-15.84	4.707548
5 (Most)	2.19	0.77	-13.76	18.14004	-4.80	0.24	-13.33	3.721612
	(Wald test of parameter: 0.725)				*(Wald test of parameter: 0.368)*			
**Continuous urban fabric**								
1 (Least)			ref				ref	
2	-1.62	0.78	-14.34	11.10	-0.34	0.92	-7.69	7.01

For peer review only

For peer review only - http://bmjopen.bmj.com/site/about/guidelines.xhtml

3	-4.22	0.59	-20.97	12.53	0.40	0.95	-14.96	15.77
4	-6.72	0.39	-23.49	10.05	-0.36	0.96	-15.72	14.99
5 (Most)	-0.93	0.87	-13.68	11.82	5.78	0.36	-7.69	19.25
	(Wald test of parameter: 0.791)				*(Wald test of parameter: 0.070)*			
**Discontinuous dense urban fabric**								
1 (Least)			ref				ref	
2	-3.51	0.51	-15.00	7.98	-0.71	0.89	-11.37	9.96
3	-2.21	0.64	-12.37	7.96	-6.60	0.13	-15.61	2.42
4	-3.91	0.47	-15.38	7.56	-7.30	0.15	-17.71	3.12
5 (Most)	3.36	0.52	-7.86	14.60	-3.33	0.53	-14.60	7.93
	(Wald test of parameter: 0.737)				*(Wald test of parameter: 0.09)*			
**Discontinuous medium density urban fabric**								
1 (Least)			ref				ref	
2	-2.18	0.64	-12.17	7.80	-2.01	0.50	-8.34	4.32
3	7.49	0.08	-1.13	16.12	7.87	0.01	2.48	13.26
4	2.02	0.74	-11.17	15.21	5.07	0.45	-9.24	19.38
5 (Most)	7.92	0.35	-10.01	25.86	1.53	0.80	-11.36	14.42
	(Wald test of parameter: 0.161)				*(Wald test of parameter: 0.083)*			
**Discontinuous low density urban fabric**								
1 (Least)			ref				ref	
2	0.67	0.92	-14.40	15.73	0.19	0.98	-18.09	18.47
3	2.83	0.63	-9.82	15.48	1.37	0.82	-12.03	14.77
4	5.38	0.39	-8.08	18.84	0.88	0.92	-17.35	19.10
5 (Most)	1.47	0.84	-13.93	16.87	-3.82	0.69	-24.54	16.89
	(Wald test of parameter: 0.822)				*(Wald test of parameter: 0.408)*			
**Discontinuous very low density urban fabric**								
1 (Least)			ref				ref	
2	12.10	0.03	1.90	22.30	3.23	0.30	-3.38	9.85
3 (Most)	0.62	0.91	-10.81	12.06	-0.38	0.93	-9.62	8.85
	(Wald test of parameter: 0.065)				*(Wald test of parameter: 0.563)*			
**Residential proportion**								
1 (Least)			ref				ref	
2	-0.70	0.92	-16.68	15.27	-6.19	0.29	-18.52	6.15
3	-4.58	0.40	-16.14	6.97	-2.68	0.64	-14.96	9.60
4	-2.50	0.61	-13.04	8.04	-5.32	0.21	-14.18	3.54
5 (Most)	3.56	0.66	-13.90	21.02	-0.70	0.90	-12.82	11.42
	(Wald test of parameter: 0.469)				*(Wald test of parameter: 0.155)*			
**Fast transit roads and associated land**								
1 (Least)			ref				ref	
2	3.08	0.61	-9.88	16.04	2.18	0.58	-6.31	10.68
3 (Most)	0.83	0.89	-12.17	13.82	2.81	0.63	-9.79	15.41
	(Wald test of parameter: 0.836)				*(Wald test of parameter: 0.846)*			

For peer review only

For peer review only - http://bmjopen.bmj.com/site/about/guidelines.xhtml

**Forests**								
1 (Least)		ref				ref		
2	-6.93	0.28	-20.41	6.55	-13.55	0.02	-24.82	-2.28
3	-5.82	0.13	-13.75	2.11	-10.43	0.00	-16.25	-4.62
4	-0.73	0.89	-12.67	11.21	-8.27	0.13	-19.36	2.83
5 (Most)	-4.99	0.20	-13.08	3.10	-15.96	0.00	-24.74	-7.17
	(Wald test of parameter: 0.309)				*(Wald test of parameter: 0.010)*			
**Green urban areas**								
1 (Least)		ref				ref		
2	1.48	0.67	-6.00	8.95	4.07	0.49	-8.47	16.62
3	-5.99	0.06	-12.37	0.39	-3.06	0.50	-12.89	6.77
4	-10.07	0.05	-19.96	-0.19	-9.26	0.17	-23.29	4.77
5 (Most)	15.37	0.02	3.66	27.08	3.85	0.50	-8.48	16.19
	(Wald test of parameter: 0.001)				*(Wald test of parameter: 0.004)*			
**Industrial, commercial, public, military**								
1 (Least)		ref				ref		
2	-12.53	0.14	-29.98	4.91	-0.92	0.91	-18.26	16.43
3	-1.70	0.81	-16.73	13.32	4.72	0.48	-9.75	19.20
4	-7.70	0.22	-20.69	5.30	-1.34	0.80	-12.77	10.08
5 (Most)	-2.81	0.51	-11.94	6.31	6.09	0.06	-0.19	12.37
	(Wald test of parameter: 0.491)				*(Wald test of parameter: 0.032)*			
**Isolated structures**								
1 (Least)		ref				ref		
2	-5.55	0.38	-19.15	8.04	-7.79	0.15	-18.77	3.19
3	-2.28	0.70	-15.09	10.53	-2.19	0.58	-10.70	6.31
4	-5.12	0.33	-16.15	5.92	-10.66	0.01	-17.95	-3.37
5 (Most)	-6.14	0.33	-19.55	7.26	-12.35	0.00	-16.73	-7.98
	(Wald test of parameter: 0.814)				*(Wald test of parameter: 0.001)*			
**Land without current use**								
1 (Least)		ref				ref		
2	0.14	0.96	-6.55	6.84	-2.02	0.43	-7.45	3.41
3	8.29	0.11	-2.13	18.71	11.30	0.01	3.43	19.18
4	14.75	0.01	5.70	23.81	12.81	0.00	6.49	19.14
5 (Most)	13.67	0.04	1.05	26.29	9.30	0.01	3.09	15.51
	(Wald test of parameter: <0.001)				*(Wald test of parameter: <0.001)*			
**Mineral extraction and dump sites**								
1 (Least)		ref				ref		
2	7.67	0.18	-4.27	19.60	5.02	0.22	-3.42	13.46
3	8.34	0.05	-0.16	16.83	6.73	0.04	0.27	13.18
4	7.86	0.14	-3.11	18.84	7.92	0.12	-2.45	18.30

For peer review only

5 (Most)	7.16	0.22	-4.90	19.23	4.74	0.20	-2.91	12.39
	(Wald test of parameter: 0.139)				*(Wald test of parameter: 0.016)*			
**Other roads and associated land**								
1 (Least)			ref				ref	
2	-8.85	0.07	-18.65	0.94	-1.47	0.61	-7.77	4.82
3	-12.31	0.03	-22.79	-1.82	-4.27	0.22	-11.59	3.05
4	-6.65	0.17	-16.66	3.35	1.69	0.42	-2.76	6.15
5 (Most)	1.52	0.82	-13.31	16.35	7.74	0.15	-3.28	18.76
	(Wald test of parameter: 0.080)				*(Wald test of parameter: 0.174)*			
**Railways and associated land**								
1 (Least)			ref				ref	
2	-3.66	0.43	-13.61	6.29	-1.69	0.61	-8.85	5.46
3	-0.19	0.97	-11.74	11.35	3.45	0.42	-5.74	12.65
4	0.12	0.99	-13.95	14.19	3.57	0.52	-8.36	15.49
5 (Most)	-3.42	0.52	-14.82	7.98	4.93	0.27	-4.39	14.26
	(Wald test of parameter: 0.883)				*(Wald test of parameter: 0.215)*			
**Sports and leisure facilities**								
1 (Least)			ref				ref	
2	-7.42	0.17	-18.66	3.82	-7.20	0.13	-16.97	2.58
3	-18.90	0.00	-27.75	-10.06	-15.15	0.04	-29.78	-0.53
4	-13.80	0.02	-25.27	-2.33	-13.98	0.06	-28.92	0.97
5 (Most)	-19.87	0.00	-31.05	-8.68	-15.16	0.06	-31.38	1.07
	(Wald test of parameter: 0.002)				*(Wald test of parameter: 0.259)*			
**Water bodies**								
1 (Least)			ref				ref	
2	1.97	0.53	-4.69	8.63	0.30	0.91	-5.51	6.10
3	3.49	0.35	-4.42	11.41	1.22	0.70	-5.72	8.16
4	9.52	0.10	-2.14	21.18	5.92	0.02	1.18	10.65
5 (Most)	7.52	0.20	-4.67	19.72	1.55	0.54	-3.90	7.00
	(Wald test of parameter: 0.474)				*(Wald test of parameter: 0.062)*			

* Due to low proportion of land area classed as airports within cities, the first three quintiles combined within the model, essentially representing less than ~0.1% of the total area.

For peer review only - http://bmjopen.bmj.com/site/about/guidelines.xhtml

BMJ Open

Which urban land covers/uses are associated with residents' mortality? A cross-sectional, ecological, pan-European study of 233 cities.

Journal:	*BMJ Open*
Manuscript ID	bmjopen-2019-033623.R1
Article Type:	Original research
Date Submitted by the Author:	21-Oct-2019
Complete List of Authors:	Olsen, Jonathan; University of Glasgow, Institute of Health and Wellbeing<br>Nicholls, Natalie; University of Glasgow, MRC/CSO Social and Public Health Sciences Unit<br>Moon, Graham; University of Southampton, Geography and Environment<br>Pearce, Jamie; University of Edinburgh, Institute of Geography and the Lived Environment<br>Shortt, Niamh; University of Edinburgh, Centre for Research on Environment, Society and Health. School of Geosciences<br>Mitchell, Richard; Glasgow University, Public Health
**Primary Subject Heading**:	Epidemiology
Secondary Subject Heading:	Public health
Keywords:	EPIDEMIOLOGY, PUBLIC HEALTH, STATISTICS & RESEARCH METHODS

SCHOLARONE™
Manuscripts

Which urban land covers/uses are associated with residents' mortality? A cross-sectional, ecological, pan-European study of 233 cities.

Jonathan R. Olsen[1], Natalie Nicholls[1], Graham Moon[2], Jamie Pearce[3], Niamh Shortt[3], Richard Mitchell[1]

[1]MRC/CSO Social and Public Health Sciences Unit, University of Glasgow, Glasgow, UK
[2]School of Geography and Environmental Sciences, University of Southampton, Southampton, UK
[3]School of Geosciences, University of Edinburgh, Edinburgh, UK

Correspondence:
Dr Jonathan R Olsen
MRC/CSO Social and Public Health Sciences Unit
University of Glasgow
200 Renfield Street
Glasgow
G2 3AX
Phone: 0141 353 7539
Email: jonathan.olsen@glasgow.ac.uk

Key words: urban; mortality; landscape; ecology; land use; land cover; urban landscape.

Tables : 2
Figures : 3
Supplementary tables : 3

For peer review only

For peer review only - http://bmjopen.bmj.com/site/about/guidelines.xhtml

Abstract

Objectives:

The study aim was to determine whether the range and distribution of all, and proportions of specific, land covers/uses within European cities are associated with the city-specific mortality rate.

Setting:

233 European cities within 24 countries.

Participants:

Aggregated city-level all-cause standardised mortality rate by gender and Western or Eastern European Region.

Results:

The proportion of specific land covers/uses within a city was related to mortality, displaying differences by macroregion and sex. The land covers/uses associated with lower SMR for both Western and Eastern European cities were those characterised by 'natural' green space, such as forests and semi-natural areas (Coef:-18.3, 95%CI -27.8 to -6.9). Dense housing was related to a higher SMR, most prominently in Western European cities (Coef:17.4, 95%CI 9.6 to 25.2).

Conclusions:

There is pressure to build on urban natural spaces, both for economic gain and because compact cities are regarded as more sustainable, yet here we offer evidence that doing so may detract from residents' health. Our study suggests that urban planners and developers need to regard retaining more wild and unstructured green space as important for healthy city systems.

Keywords: urban; mortality; landscape; ecology; land use; land cover; urban landscape.

Article summary

Strengths and limitations of this study

- This was a large international study of 233 cities within 24 countries.
- We used high quality, internationally comparable land cover/use data from the European Urban Atlas.
- We were able to examine the impact of land covers/uses both collectively (via landscape metrics) and individually.
- Previously validated city-level SMR data were spatially joined with contemporaneous land cover/use data.
- Although associations between land covers/uses and SMRs were described, we have no information on how the individuals used or were exposed to the land covers/uses.

For peer review only - http://bmjopen.bmj.com/site/about/guidelines.xhtml

Background

In 2014, 54% of the world's population lived in urban environments; this is set to rise to 66% by 2050[1]. It is therefore important that urban environments are designed to enable and promote good health. Urban areas are complex systems and one emergent feature is the health of their residents[2]. There are stark between-city inequalities in population health[3] and previous work on trends and inequalities between European cities in sex-specific mortality rates suggests that a city environment itself, is an important influence[4-6].

The physical extent, design and composition of cities are important components of the urban system and are associated with the health and wellbeing of residents[7-9]. The diverse development trajectories of cities means there is considerable variety in their size, configuration, and content[10]. This variety provides a rich natural laboratory to study the implications for health of their design and construction. Given that most cities are subject to some kind of planning control and vision, there is also great potential for epidemiological insights into how the urban landscape affects health to impact on the lived experiences of billions of people.

This study is about 'what is on the ground' in a city, which we refer to as land cover/uses. Land cover refers to what the surface actually is, for example forests, concrete or water. Land uses are the purposes for which humans use the land, such as; airports or roads. Urban land cover/use can influence health via multiple mechanisms including the promotion or inhibiting of healthy behaviours[11], increasing exposure to pathogens such as air pollutants and noise[12], or offering psychologically restorative spaces[13].

Evidence suggests that land cover/use exerts an independent influence on residents, over and above residents' own characteristics. For example the density of settlement and transport networks have been associated with variation in mortality risk across a number of English cities[14]. In a previous multi-city European study, we found that the presence and quantity of specific land cover/uses within a city was associated with residents' reported life satisfaction. We also found that specific land covers/uses, and a more even distribution of the land covers/uses (as opposed to dominance by one or two land covers/uses), were both associated with lower levels of socio-economic inequality in life

For peer review only - http://bmjopen.bmj.com/site/about/guidelines.xhtml

satisfaction within the city[15]. We hypothesised that the range and distribution of land covers/uses speaks to the range of opportunities and environments, and hence affordances[16] that the city provides – and that this might explain the associations with life satisfaction levels and its equality. However, life satisfaction is fundamentally a subjective measure of well-being, and may be particularly sensitive to the environment. Therefore in this study we focused on mortality which is, arguably, a more robust measure of population health. In doing so, we were able to include a substantially larger sample of European cities (n=233). No previous study has assessed associations between city-level land covers/uses and mortality in such a large and diverse international sample of cities.

The study aim was to determine whether the range and distribution of all, and proportions of specific, land covers/uses within European cities is associated with the city-specific mortality rate. Previous research found that a city's wider macroregion (i.e. its location in Eastern or Western Europe) was an important determinant of mortality rate, and moderator of association between environment and health[5 17], therefore this was also explored. Our specific research questions were:

i) Is the range and distribution of land covers/uses within a city associated with city-level mortality rates?

ii) Which, if any, specific land cover/uses are associated with city-level mortality rates?

iii) How do these between associations vary with sex and by macroregion?

For peer review only - http://bmjopen.bmj.com/site/about/guidelines.xhtml

Methods

Setting and data sources

European Urban Atlas

The 2006 Urban Atlas provided pan-European comparable land cover/use data collected and digitised from satellite data for urban areas[18]. We selected data for this period to match the time period for the available mortality data. The Atlas distinguishes 26 different land cover/use categories at a $10m^2$ resolution[19]. Figure 1 provides an illustration of these data for part of one city. In this example, 20 different land uses are shown and labelled. Full land use descriptions are available in Supplementary Table 1. The land cover/use data were matched to the same city administrative boundaries as the mortality data.

Geoprocessing of Urban Atlas data

We calculated the total area (km^2) for each land cover/use by city, using an extension for ArcMap GIS called Patch Analyst[20]. This was then converted to proportion of land cover/use by city (some values equalled zero if the land cover/use was not present in a city, for example, not all cities contained an airport). Following our previous work[15], we then calculated landscape metrics. These are quantitative summaries of the range and statistical distribution of the various land covers/uses within a whole city. We calculated Shannon's diversity index (SDI) and Shannon's Evenness index (SEI)[21] to assess the variety of all land covers/uses, and their relative availability, within each city. Only land covers/uses present within each city were included, this provided a relative measure of the diversity of land cover/uses available at the city level (SDI) and the distribution of area among the land cover/uses present within a city (SEI).

Gross Domestic Product (GDP)

Following Richardson *et al.* (2017)[3], the GDP per 1000 capita for the city region was used as a measure of affluence/poverty for that city. Historic GDP per country for the years 2003-2006 inclusive were extracted from Eurostat and averaged to provide a single measure for each city.

European mortality data (Standardised Mortality Ratios)

Mortality was measured using all-cause and age-group standardised mortality ratios (SMR) for males and females separately, calculated for 233 European cities by Richardson et al[3]. Values higher than

For peer review only - http://bmjopen.bmj.com/site/about/guidelines.xhtml

100 indicated a higher level of mortality than expected given the time period and age structure of the city population, and values lower than 100 indicated a lower level. Richardson et al's SMR data covered the time period 1999 to 2009, divided into three waves; the second of which was 2003 to 2006. This time period most closely matched the date of the land cover measures from the Urban Atlas. The mortality indicator was at the city-level. No information was available about mortality rates either for socio-economic groups or spatial locations within the city. We could not, therefore, assess within-city inequalities in this study.

The SMR, land cover/use and GDP data were matched at city level. The final dataset included 233 cities (within 24 countries). The cities were allocated to either Western or Eastern-Central bloc macroregion, following Richardson et al[3], referred to hereafter as Western or Eastern. There were 85 cities (in 11 countries) classed as Eastern.

Patient and public involvement

No patients were involved in the development of the research question, design and implementation of the study, or interpretation of the results.

Statistical Analysis

First, linear regression models assessed associations between the landscape metrics and city SMR data, adjusting for city GDP. Next, models for specific land cover/uses were built. Preliminary analyses suggested that associations between the specific land cover/use measures and SMR were not linear, so the former were categorised. To do this, the proportion of the total area covered by each land measure was calculated within each city, and then classified into quintiles. This process was carried out separately for each macroregion. We categorised the data into quintiles rather than apply polynomial terms in order to aid interpretation and therefore help guide policymakers[22]. Further, this approach also allowed us to generate the marginal estimates, which were plotted. These quintiles were then fit as factors, along with city GDP, in a linear regression, while also allowing for clustering within country. Each land measure was assessed separately using Wald statistics and subjected to post-hoc pairwise comparisons using Sidak's correction for multiple testing across all terms[23]. We chose not to include all land cover/uses in a singular model due to concern of

For peer review only - http://bmjopen.bmj.com/site/about/guidelines.xhtml

multicollinearity, which was confirmed when tested (Eastern European Region – VIF: 17.20, Western European Cities – VIF: 6.41)[24 25]. All analyses were run separately by sex and macroregion. Stata(SE,14)[26] and R(v3.6.0)[27] were used for analysis, with significance levels set at 5%.

Results

For the models looking at specific land covers/uses we present our results in three ways. First, we highlight the land covers/uses for which we found strongest evidence of association with either lower or higher mortality rates (Table 1). This selection was systematic, based on Wald test values and on both size and apparent 'dose response' of associations between quintiles of land cover/use and mortality. Table 1 crudely 'ranks' these selected land covers/uses according to the extent to which they were associated with mortality in both macroregions, and both sexes (which we call 'universal'). A downward pointing arrow denotes that more of that land cover/use was associated with lower mortality, and an upward arrow that it was associated with higher mortality. Second, we present regression coefficients for these selected land cover/uses (Table 2). Supplementary table 2 (Western Europe) and supplementary table 3 (Eastern Europe) provide the full set of regression coefficients for *all* land cover/uses, together with Wald statistics. Third, we graph marginal means from the adjusted regression models for associations identified in Table 1 as being either ***universal*** or ***partially universal*** (Figures 2 and 3). These figures convert the associations to estimated SMRs, perhaps a more meaningful expression of the results. The reader is invited to pay attention to the position on the Y axis, and slope, of the lines in the figures. We found no meaningful associations between SMRs and the landscape metrics and therefore do not report those results.

For peer review only - http://bmjopen.bmj.com/site/about/guidelines.xhtml

Table 1: Land cover/uses with strongest and most consistent evidence of association with city SMRs

**Universal (Both regions and sexes)**			
**West (M&F)**	***Trend***	**East (M&F)**	***Trend***
Agricultural, semi-natural areas, wetlands	↓ *SMR*	Agricultural, semi-natural areas, wetlands	↓ *SMR*
**Partially universal (across either both regions and/or sexes)**			
**West F only**	***Trend***	**East (M&F)**	***Trend***
Green urban areas	↑ *SMR*	Green urban areas	↑ *SMR*
**West (M&F)**	***Trend***	**East F only**	***Trend***
Industrial, commercial, public, military	↑ *SMR*	Industrial, commercial, public, military	↑ *SMR*
**Regional (Both sexes)**			
**West (M&F)**	***Trend***	**East (M&F)**	***Trend***
Discontinuous low density urban fabric*	↑ *SMR*		
Residential*	↑ *SMR*		
		Sports and leisure facilities	↓ *SMR*
		Land without current use	↑ *SMR*
**Sex (both regions)**			
**Males**	***Trend***	**Females (E & W)**	***Trend***
		Forests	↓ *SMR*
**Regions & opposite sex**			
**West M**	***Trend***	**East F**	***Trend***
Isolated structures	↓ *SMR*	Isolated structures	↓ *SMR*

* Most evident in cities with the highest proportion of this land cover/use (quintile 5)

eview only

For peer review only - http://bmjopen.bmj.com/site/about/guidelines.xhtml

Table 2: Regression coefficients of land cover/uses found to contribute to a model for a particular macroregion/sex based on Wald test measure (significant results marked (<0.05) in bold font).

Quintiles (proportion of land-use)	West								East							
	Males				Females				Males				Females			
	Coef	LL 95% CI	UL 95% CI	p	Coef	LL 95% CI	UL 95% CI	p	Coef	LL 95% CI	UL 95% CI	p	Coef	LL 95% CI	UL 95% CI	p
**Agricultural, semi-natural areas, wetlands**																
1	ref				ref				ref				ref			
2	-10.22	-17.69	-2.75	0.011	**-11.66**	**-20.75**	**-2.58**	**0.016**	**-14.45**	**-30.34**	**1.44**	**0.07**	-3.68	-12.24	4.88	0.36
3	**-11.67**	**-17.30**	**-6.04**	**0.001**	**-13.03**	**-23.12**	**-2.94**	**0.015**	-11.51	-30.34	7.32	0.203	-2.86	-10.81	5.08	0.441
4	**-12.63**	**-19.14**	**-6.13**	**0.001**	**-19.71**	**-33.78**	**-5.65**	**0.01**	**-18.67**	**-37.03**	**-0.32**	**0.047**	-7.14	-16.67	2.39	0.126
5	**-13.66**	**-21.08**	**-6.23**	**0.002**	**-18.32**	**-29.77**	**-6.88**	**0.004**	-3.89	-26.56	18.78	0.71	5.76	-8.51	20.03	0.39
	(Wald test of parameter: 0.006)				*(Wald test of parameter: 0.051)*				*(Wald test of parameter: 0.017)*				*(Wald test of parameter: 0.014)*			
**Forests**																
1	ref				ref				ref				ref			
2	-4.60	-10.36	1.16	0.11	**-9.71**	**-18.44**	**-0.98**	**0.03**	-6.93	-20.41	6.55	0.28	**-13.55**	**-24.82**	**-2.28**	**0.02**
3	-4.53	-10.66	1.60	0.13	-8.48	-18.81	1.85	0.10	-5.82	-13.75	2.11	0.13	**-10.43**	**-16.25**	**-4.62**	**0.00**
4	**-9.54**	**-19.44**	**0.37**	**0.06**	**-15.13**	**-31.88**	**1.62**	**0.07**	-0.73	-12.67	11.21	0.89	-8.27	-19.36	2.83	0.13
5	**-9.03**	**-16.99**	**-1.08**	**0.03**	**-12.59**	**-25.08**	**-0.11**	**0.05**	-4.99	-13.08	3.10	0.20	**-15.96**	**-24.74**	**-7.17**	**0.00**
	(Wald test of parameter: 0.156)				*(Wald test of parameter: 0.005)*				*(Wald test of parameter: 0.309)*				*(Wald test of parameter: 0.010)*			
**Green urban areas**																
1	ref				ref				ref				ref			
2	1.60	-4.47	7.66	0.58	-0.02	-7.11	7.07	1.00	1.48	-6.00	8.95	0.67	4.07	-8.47	16.62	0.49
3	3.96	-2.05	9.96	0.18	1.43	-7.81	10.67	0.74	**-5.99**	**-12.37**	**0.39**	**0.06**	-3.06	-12.89	6.77	0.50
4	**7.23**	**-0.26**	**14.73**	**0.06**	**9.57**	**1.21**	**17.93**	**0.03**	**-10.07**	**-19.96**	**-0.19**	**0.05**	-9.26	-23.29	4.77	0.17
5	**11.33**	**2.44**	**20.21**	**0.02**	**15.06**	**4.45**	**25.67**	**0.01**	**15.37**	**3.66**	**27.08**	**0.02**	3.85	-8.48	16.19	0.50
	(Wald test of parameter: 0.132)				*(Wald test of parameter: 0.029)*				*(Wald test of parameter: 0.001)*				*(Wald test of parameter: 0.004)*			
**Industrial, commercial, public, military**																
1	ref				ref				ref				ref			
2	2.02	-2.51	6.55	0.35	-0.23	-6.54	6.08	0.94	-12.53	-29.98	4.91	0.14	-0.92	-18.26	16.43	0.91
3	1.34	-5.24	7.93	0.67	1.75	-6.93	10.42	0.67	-1.70	-16.73	13.32	0.81	4.72	-9.75	19.20	0.48
4	3.24	-2.16	8.65	0.22	**6.76**	**2.31**	**11.21**	**0.01**	-7.70	-20.69	5.30	0.22	-1.34	-12.77	10.08	0.80

For peer review only - http://bmjopen.bmj.com/site/about/guidelines.xhtml

5	**10.64**	**3.79**	**17.50**	**0.01**	**11.02**	**0.93**	**21.11**	**0.04**	-2.81	-11.94	6.31	0.51	6.09	-0.19	12.37	0.06
	(Wald test of parameter: 0.049)				*(Wald test of parameter: 0.023)*				*(Wald test of parameter: 0.491)*				*(Wald test of parameter: 0.033)*			
**Isolated structures**																
1	ref				ref				ref				ref			
2	-5.04	-12.40	2.32	0.16	**-9.40**	**-19.77**	**0.97**	**0.07**	-5.55	-19.15	8.04	0.38	-7.79	-18.77	3.19	0.15
					**-**											
3	**-6.42**	**-10.46**	**-2.39**	**0.00**	**10.60**	**-18.98**	**-2.23**	**0.02**	-2.28	-15.09	10.53	0.70	-2.19	-10.70	6.31	0.58
4	**-7.94**	**-14.52**	**-1.35**	**0.02**	**-7.23**	**-14.35**	**-0.12**	**0.05**	-5.12	-16.15	5.92	0.33	**-10.66**	**-17.95**	**-3.37**	**0.01**
5	**-7.87**	**-15.28**	**-0.46**	**0.04**	-8.46	-18.68	1.77	0.10	-6.14	-19.55	7.26	0.33	**-12.35**	**-16.73**	**-7.98**	**<0.001**
	(Wald test of parameter: 0.029)				*(Wald test of parameter: 0.145)*				*(Wald test of parameter: 0.814)*				*(Wald test of parameter: 0.001)*			
**Residential proportion**																
1	ref				ref				ref				ref			
2	1.13	-5.96	8.22	0.74	0.82	-13.05	14.68	0.90	-0.70	-16.68	15.27	15.27	-6.19	-18.52	6.15	0.29
3	2.77	-2.63	8.17	0.29	0.23	-9.54	10.00	0.96	-4.58	-16.14	6.97	6.97	-2.68	-14.96	9.60	0.64
4	3.87	-0.53	8.26	0.08	3.86	-0.96	8.68	0.11	-2.50	-13.04	8.04	8.04	-5.32	-14.18	3.54	0.21
5	**13.19**	**7.88**	**18.49**	**<0.001**	**17.39**	**9.61**	**25.18**	**<0.001**	3.56	-13.90	21.02	21.02	-0.70	-12.82	11.42	0.90
	(Wald test of parameter: <0.001)				*(Wald test of parameter: 0.004)*				*(Wald test of parameter: 0.469)*				*(Wald test of parameter: 0.155)*			
**Land without current use**																
1 (Least)	ref				ref				ref				ref			
2	0.65	-4.57	5.88	0.79	-4.16	-14.34	6.03	0.39	0.14	-6.55	6.84	0.96	-2.02	-7.45	3.41	0.43
3	1.09	-4.36	6.55	0.67	-4.20	-12.08	3.69	0.27	8.29	-2.13	18.71	0.11	**11.30**	**3.43**	**19.18**	**0.01**
4	0.98	-3.80	5.77	0.67	-1.62	-5.91	2.67	0.43	**14.75**	**5.70**	**23.81**	**0.01**	**12.81**	**6.49**	**19.14**	**0.00**
5 (Most)	**10.18**	**0.07**	**20.30**	**0.05**	4.58	-6.12	15.28	0.37	**13.67**	**1.05**	**26.29**	**0.04**	**9.30**	**3.09**	**15.51**	**0.01**
	(Wald test of parameter: 0.249)				*(Wald test of parameter: 0.316)*				*(Wald test of parameter: <0.001)*				*(Wald test of parameter: <0.001)*			
**Discontinuous low density urban fabric**																
1 (Least)	ref				ref				ref				ref			
2	0.46	-6.48	7.40	0.89	-3.85	-13.57	5.87	0.41	0.67	-14.40	15.73	0.92	0.19	-18.09	18.47	0.98
3	-0.40	-6.95	6.16	0.90	-5.65	-17.07	5.76	0.30	2.83	-9.82	15.48	0.63	1.37	-12.03	14.77	0.82
4	-0.58	-12.46	11.31	0.92	-6.61	-27.00	13.79	0.50	5.38	-8.08	18.84	0.39	0.88	-17.35	19.10	0.92
5 (Most)	**7.98**	**2.22**	**13.75**	**0.01**	**7.92**	**-1.62**	**17.46**	**0.10**	1.47	-13.93	16.87	0.84	-3.82	-24.54	16.89	0.69
	(Wald test of parameter: 0.005)				*(Wald test of parameter: 0.027)*				*(Wald test of parameter: 0.822)*				*(Wald test of parameter: 0.408)*			
**Sports and leisure facilities**																
1 (Least)	ref				ref				Ref				ref			
2	1.68	-6.25	9.61	0.65	-4.11	-20.19	11.97	0.59	-7.42	-18.66	3.82	0.17	-7.20	-16.97	2.58	0.13
3	2.48	-3.15	8.12	0.36	0.01	-10.95	10.97	1.00	**-18.90**	**-27.75**	**-10.06**	**<0.001**	**-15.15**	**-29.78**	**-0.53**	**0.04**
4	4.60	-0.11	9.31	0.06	**9.67**	**1.18**	**18.16**	**0.03**	**-13.80**	**-25.27**	**-2.33**	**0.020**	-13.98	-28.92	0.97	0.06
5 (Most)	**10.39**	**1.82**	**18.95**	**0.02**	**14.12**	**2.88**	**25.35**	**0.02**	**-19.87**	**-31.05**	**-8.68**	**<0.001**	-15.16	-31.38	1.07	0.06
	(Wald test of parameter: 0.179)				*(Wald test of parameter: 0.086)*				*(Wald test of parameter: 0.002)*				*(Wald test of parameter: 0.259)*			

Land cover/uses associated with lower mortality

In general, cities with a higher proportion of ***agricultural, semi-natural areas and wetlands*** enjoyed lower mortality rates (Figure 2). In Eastern cities this advantage weakened in cities with the *highest* proportion of this land-use (Figure 2, Table 2). Figure 2 shows a somewhat large difference in SMR between both sexes for Western cities in quintile 1 and quintile 5. A higher proportion of ***forests*** within a city was also associated with lower SMR for females in both Eastern and Western European cities, but not males (Table 2). In Western cities, the proportion of ***isolated structures*** *(single dwellings surrounded on all sides with natural green land)* was associated with lower SMRs for males, but in Eastern cities this protective association was present for females only (Table 2). For Eastern European cities only, higher proportion of land containing ***sports and leisure facilities*** was associated with lower SMRs, though only significantly for men (Table 2).

Land cover/uses associated with higher mortality

The two land cover/uses most consistently associated with higher mortality were **industrial, commercial, public and military**, and **green urban areas** (figure 3). In Western European cities higher proportions of the former were associated with higher SMRs, with a sharpening of association in cities containing the most (quintile 5). The association was not consistent or significant for men living in Eastern cities but was for women, though the trend fluctuated and only cities with greatest proportions of this land cover/use were associated with a higher SMR. A positive association between **green urban areas** and SMR was seen for Western women, and both sexes in Eastern cities (Table 2, Figure 3). The trend in Eastern cities suggested a threshold effect that may be substantially more important for health, with modest reductions in SMR as proportions increase from quintiles 1 to 4, and then a sudden and steep adverse association at quintile 5.

More derelict land (***land without current use***) was associated with higher SMRs in the Eastern cities, with the greatest adverse association quintiles 4 and 5. Western European cities with higher proportions of land classified as ***residential*** and ***discontinuous low density urban fabric*** had

For peer review only - http://bmjopen.bmj.com/site/about/guidelines.xhtml

higher SMRs, particularly for cities in quintile 5 (Table 2) , suggesting a critical level at which these land cover/uses become substantially more important to health.

For peer review only

For peer review only - http://bmjopen.bmj.com/site/about/guidelines.xhtml

Discussion

This is the first pan-European study to examine whether, and which, land cover/uses within a whole city are associated with mortality rates. By integrating health and environmental data from a large number of cities across Western and Eastern Europe we found that: the proportion of some specific land covers/uses within a city was related to mortality; most land covers/uses were not clearly associated with mortality and; there were differences by macroregion and sex in association. We found no evidence that the overall distribution and balance of multiple land cover/uses, measured by landscape metrics, was related to mortality rates.

Overall, we observed that higher proportions of natural spaces and less dense or non-residential land cover/use was associated with lower mortality. For example, greater proportions of lower density settlement (such as **isolated structures**) showed some association with lower SMR, whilst SMRs were higher for Western European cities with a greater residential proportion and low density discontinuous cover (which is principally housing). Specifically, our most consistent finding was that presence of more 'relatively wild' green spaces, such as **agricultural**, **semi-natural areas and wetlands** and **forests** was associated with lower SMRs, an association observed across sexes and macroregions and relatively strongly. The health benefits of contact with nature are very well established from both experimental and observational studies[28]. These kinds of spaces may be particularly restorative or conducive of leisure and recreation. Studies have shown the protective effects of natural environments and association with reduced risk of mortality. For example two large Canadian studies examined green space using normalized difference vegetation index (NDVI) around residents' home and showed a higher NDVI index was associated with lower mortality rates[29 30]. Our results for the more natural spaces are in stark contrast to those for **green urban areas**, the proportion of which was relatively consistently associated with *higher* mortality. At first, this seems like a directly contradictory finding, but it is important to be clear what kinds of spaces are classified as 'green urban areas' in the Urban Atlas. Large urban parks are not classified solely as 'green urban areas' and will be separated into their designated components, for example if a park contains a botanical garden, zoo or national trust gardens, these areas will be classified as an Industrial or

For peer review only - http://bmjopen.bmj.com/site/about/guidelines.xhtml

Commercial areas; blue spaces within parks will be defined as waters; any space which can be used for sport or containing sport facilities (goal posts, cricket or basketball markings,) will be defined as sports or leisure facilities. In this land use classification, ‘green urban areas’ are relatively small and manicured ‘in-fill’ green spaces within residential and commercial developments. Their association with greater mortality is not unprecedented in the literature. Previous work found that the total amount of green space within American cities was associated with higher mortality rates, suggesting that the indicator was capturing sprawling cities, however this study was unable to distinguish between the green space type[31]. In light of our findings, the American study may have been capturing the development of semi-natural areas into ‘in-fill’ green developments. We have previously found that greater amounts of ‘**green urban areas**’ in European cities were associated with reduced reported life satisfaction for its city residents[15]. The salutogenic effects of nature may be maximised by more natural areas and / or the ‘pockets of green’ in and among urban developments may be insufficient to offset the adversity of living in dense residential and commercial environments.

There were some differences between region and sex in the direction of associations. We found that less desirable land types/uses within cities, such as **land without use**, were associated with higher SMRs for those living in Eastern European cities. Cities depend on their residents for economic, social, cultural and environmental prosperity and maintaining a diverse, skilled, and satisfied residential population is vital for a city since their disenchantment could trigger a vicious downward spiral[32]. Dereliction of cities has been linked to decreasing employment rates due to many individuals, particularly younger and educated, migrating from these areas to more prosperous cities[33] and individuals living in areas with a high proportion of brownfield land are significantly more likely to suffer from poorer health than those with a lower proportion[34]. Although derelict and vacant land covers/uses remain in both Western and Eastern European regions and was a national problem in many Western cities during the mid/late 20th century as many industries declined or closed[33], there is country specific evidence that areas of these land cover/uses are decreasing. For example in Scotland from 2017 to 2018 derelict land decreased by 6% nationally[35], which may reduce the importance for population health. Perhaps the approaches taken by some Western governments[36] [37] to tackle it and spearhead regeneration in these areas for example, have mitigated impacts.

For peer review only - http://bmjopen.bmj.com/site/about/guidelines.xhtml

Further, the impact of **industrial, commercial, public and military** spaces within a city seemed benign for men living in Eastern European Cities but not for women, and both sexes in Western European cities. Males and females in eastern cities also seemed to benefit from greater proportions of **sports and leisure facilities**. The western / eastern differences may reflect levels of city and economic development not adequately captured by our control for GDP. Perhaps, for example, a city in the east that has substantial amounts of land dedicated to **sports and leisure facilities** hosts a population with sufficient affluence to sustain them.

Overall, our results present a challenge to healthy urban planning. Building on natural green spaces can address housing shortages, increase the local taxation base and support the development of local infrastructure (school, transportation etc.). Residential settlements with ‘green views’ command a premium price. Further, a central message of contemporary urban planning is that dense and/or compact cities are ‘sustainable’. Yet, the literature already hints that compact cities characterised by high density residential areas have both benefits and disadvantages for their residents. The environmental benefits, owing to the reduced carbon emissions required in intraurban transport and service, have been well described[4], and yet at the same time result in a reduction in quality of life measures[38]. Our findings add to this debate by suggesting that retaining more wild and unstructured green space within cities is important for health.

Strengths and weaknesses

This was a large international study of 233 cities within 24 countries. We were able to utilise high quality, internationally comparable land cover/use data from the European Urban Atlas for each city to assess the configuration and quantity of these within each city. By including a large number of cities and countries with differing economic position, we believe our results can be generalised to similar cities globally. We were able to dissect the impact of land covers/uses both collectively (via landscape metrics) and individually. We surpass some previously research by including a very large range of land covers/uses, rather than studying the impact of just one or two[39]. We were able to match previously validated SMR data to land cover/use data for the same areas and a close time point. SMRs are robust, valid and widely used measures of population health. It is important to

For peer review only

consider that land cover/uses within cities will co-occur, for example dense compact cities may have a high proportion of both industrial and high density residential land covers/uses. A strength of our analysis was that we assessed each land cover/use separately, allowing us to examine whether if similar types of land cover/uses were important for health. The aim of our study was not to understand the influence or configuration of combinations of land cover/use on health but nonetheless this offers an important line of enquiry for future research.

However, although we described associations between land covers/uses and SMRs, we have no information on how the individuals used or were exposed to the land covers/uses that we included in our analysis, for example if they live in a part of the city without that particular land use, and were unable to distinguish between land covers and land uses. This may be most pertinent for the most socio-economically deprived individuals who may have a smaller activity space and therefore the affordances of the city wide environment on their mortality may be limited. Data were aggregated to the city level and therefore may be subjected to both the ecological fallacy and modifiable areal unit problem. The development of the sample cities may also have changed considerably during individuals' life courses and the individual may not have always resided in that city, thus impacting on SMRs. As this was an ecological study and only measured land cover/use at one point in time and therefore unable to determine causality. We explored the possible effects of cultural and economic differences in land covers/uses by analysing our cities by macroregion, however we did not explore the different cultural meanings for each city to be able to ascertain if culturally affirming landscapes were health beneficial. The mortality indicator we used for the study was a city-level population level measure and therefore we were unable to explore inequality in mortality by socio-economic status.

Conclusion

We found that the proportion of specific land cover/uses within cities is associated with mortality rates there, but that these associations varied by macroregion and by sex. There is great pressure to build on wild green space for economic gain and sustainability, and to promote compact cities as sustainable. Our study suggests that urban planners and developers need to regard retaining more

For peer review only - http://bmjopen.bmj.com/site/about/guidelines.xhtml

wild and unstructured green space as important for healthy city systems, and that they should reconsider the push for dense and compact cities.

Figure 1: Urban Atlas land use/cover categories and satellite base map.

Figure 2: Estimated SMRs for cities with varying quintiles of Agricultural, semi-natural areas, wetlands (quintile 1 = lowest proportion)

Figure 3: Comparisons between SMR and quintiles land cover/uses for Green urban areas and Industrial, commercial, public, military (partially universal importance by macroregion and/or sex).

For peer review only

Contributors: JRO, NN and RM contributed to the conception and design of the study. NN performed the statistical analysis. JRO performed the geospatial analysis. RM, GM, JP & NS were involved in previous work developing the main outcome measure. JRO prepared the first draft of the manuscript, with all authors contributing to its main content and revising it with critical comments. All authors have read and approved the manuscript prior to submission, and agree to be accountable for all aspects of the work in ensuring that questions related to the accuracy or integrity of any part of the work are appropriately investigated and resolved.

Funding statement: JO, NN and RM are employed by the University of Glasgow and funded as part of the Neighbourhoods and Communities Programme (MC_UU_12017/10) (SPHSU10) at the MRC/CSO Social and Public Health Sciences Unit (SPHSU).

Competing interests: None declared.

Data sharing statement*:* Standardised Mortality Ratio data are available by emailing richard.mitchell@glasgow.ac.uk. European Urban Atlas data are freely available from the European Environment Agency (https://www.eea.europa.eu/data-and-maps/data/urban-atlas), provided by the European Commission of the European Union.

Patient consent for publication: None required.

References

1. United Nations. World Urbanization Prospects: The 2014 Revision, Highlights. Department of Economic and Social Affairs. *Population Division, United Nations* 2014
2. Frumkin H. Urban sprawl and public health. *Public Health Rep* 2002;117(3):201-17.
3. Richardson EA, Moon G, Pearce J, et al. Multi-scalar influences on mortality change over time in 274 European cities. *Soc Sci Med* 2017;179:45-51.
4. Jabareen YR. Sustainable urban forms: Their typologies, models, and concepts. *J Plan Educ Res* 2006;26(1):38-52.
5. Marí-Dell'Olmo M, Gotsens M, Palència L, et al. Socioeconomic inequalities in cause-specific mortality in 15 European cities. *J Epidemiol Community Health* 2015;69(5):432-41.
6. Gotsens M, Marí-Dell'Olmo M, Pérez K, et al. Socioeconomic inequalities in injury mortality in small areas of 15 European cities. *Health Place* 2013;24:165-72.
7. Ewing R, Schmid T, Killingsworth R, et al. Relationship between urban sprawl and physical activity, obesity, and morbidity. Urban Ecology: Springer 2008:567-82.
8. Sallis JF, Floyd MF, Rodríguez DA, et al. Role of built environments in physical activity, obesity, and cardiovascular disease. *Circulation* 2012;125(5):729-37.
9. Sarkar C, Webster C, Gallacher J. Healthy cities: public health through urban planning: Edward Elgar Publishing 2014.
10. Lawrence RJ. Human Ecology in the Context of Urbanisation. In: Nieuwenhuijsen M, Khreis H, eds. Integrating Human Health into Urban and Transport Planning. Cham, Switzerland: Springer 2018.
11. Giles-Corti B, Vernez-Moudon A, Reis R, et al. City planning and population health: a global challenge. *Lancet* 2016;388(10062):2912-24.
12. Dadvand P, Ostro B, Figueras F, et al. Residential proximity to major roads and term low birth weight: the roles of air pollution, heat, noise, and road-adjacent trees. *Epidemiology* 2014;25(4):518-25.
13. Hunter R, Cleland C, Cleary A, et al. Environmental, health, wellbeing, social and equity effects of urban green space interventions: A meta-narrative evidence synthesis. *Environ Int* 2019;130:104923.
14. Fecht D, Fortunato L, Morley D, et al. Associations between urban metrics and mortality rates in England. *Environ Health* 2016;15(1):S34.
15. Olsen JR, Nicholls N, Mitchell R. Are urban landscapes associated with reported life satisfaction and inequalities in life satisfaction at the city level? A cross-sectional study of 66 European Cities *Soc Sci Med* 2018
16. Lennon M, Douglas O, Scott M. Urban green space for health and well-being: developing an 'affordances' framework for planning and design. *J Urban Des* 2017;22(6):778-95.
17. Marmot M, Allen J, Bell R, et al. WHO European review of social determinants of health and the health divide. *Lancet* 2012;380(9846):1011-29.
18. Copernicus. Urban Atlas 2018 [Available from: https://land.copernicus.eu/local/urban-atlas accessed 11/01/2018 2018.
19. European Commission. Mapping guide for a European urban atlas: European Commission; 2012 [Available from: http://ec.europa.eu/regional_policy/sources/tender/pdf/2012066/annexe2.pdf accessed 11/01/2018 2018.
20. Patch Analyst and Patch Grid [program]. Centre for Northern Forest Ecosystem Research, Thunder Bay, Ontario.: Ontario Ministry of Natural Resources, 2012.
21. Rempel R, Carr A, Elkie P. Patch analyst for ArcGIS®. *Centre for Northern Forest Ecosystem Research, Ontario Ministry of Natural Resources Lakehead University* 2008
22. Barrio I, Arostegui I, Rodríguez-Álvarez M-X, et al. A new approach to categorising continuous variables in prediction models: Proposal and validation. *Stat Methods Med Res* 2017;26(6):2586-602.
23. Abdi H. Bonferroni and Šidák corrections for multiple comparisons In: Salkind NJ, ed. Encyclopedia of measurement and statistics. Thousand Oaks (CA):: Sage 2007.
24. Multicollinearity and regression analysis. J Phys; 2017. IOP Publishing.
25. Akinwande MO, Dikko HG, Samson A. Variance inflation factor: as a condition for the inclusion of suppressor variable (s) in regression analysis. *Open J Stat* 2015;5(07):754.

For peer review only - http://bmjopen.bmj.com/site/about/guidelines.xhtml

26. Stata Statistical Software: Release 14. [program]. College Station, TX: StataCorp LP: StataCorp., 2015.
27. R: A language and environment for statistical computing. [program]: R Foundation for Statistical Computing, 2018.
28. Hartig T, Mitchell R, De Vries S, et al. Nature and health. *Annu Rev Public Health* 2014;35:207-28.
29. Crouse DL, Pinault L, Balram A, et al. Urban greenness and mortality in Canada's largest cities: a national cohort study. *Lancet Planet Health* 2017;1(7):e289-e97.
30. Villeneuve PJ, Jerrett M, Su JG, et al. A cohort study relating urban green space with mortality in Ontario, Canada. *Environ Res* 2012;115:51-58.
31. Richardson EA, Mitchell R, Hartig T, et al. Green cities and health: a question of scale? *J Epidemiol Community Health* 2012;66(2):160-65.
32. Insch A. Branding the city as an attractive place to live. City Branding: Springer 2011:8-14.
33. Ward W. Environmental Improvements in Relation to Derelict Land. *J R Soc Health* 1970;90(4):208-11.
34. Bambra C, Robertson S, Kasim A, et al. Healthy land? An examination of the area-level association between brownfield land and morbidity and mortality in England. *Environ Plan A* 2014;46(2):433-54.
35. Scottish Goverment. Scottish Vacant and Derelict Land Survey: 2018 Edinburgh, Scotland.: Scottish Government; 2019 [Available from: https://www.gov.scot/publications/scottish-vacant-derelict-land-survey-2018/ accessed 27/09/2019 2019.
36. Northern Ireland Executive. Urban Development Grant Scheme 2019 [Available from: https://www.communities-ni.gov.uk/articles/urban-development-grant-scheme accessed 29.04.2019 2019.
37. Scottish Goverment. Capital investment for regeneration Edinburgh, Scotland: Scottish Government; 2019 [Available from: https://www.gov.scot/policies/regeneration/capital-investment/ accessed 29.04.2019 2019.
38. Neuman M. The compact city fallacy. *J Plan Educ Res* 2005;25(1):11-26.
39. Litman T, Steele R. Land use impacts on transport: How land use factors affect travel behavior: Victoria Transport Policy Institute Victoria, BC., Canada, 2012.

For peer review only

For peer review only - http://bmjopen.bmj.com/site/about/guidelines.xhtml

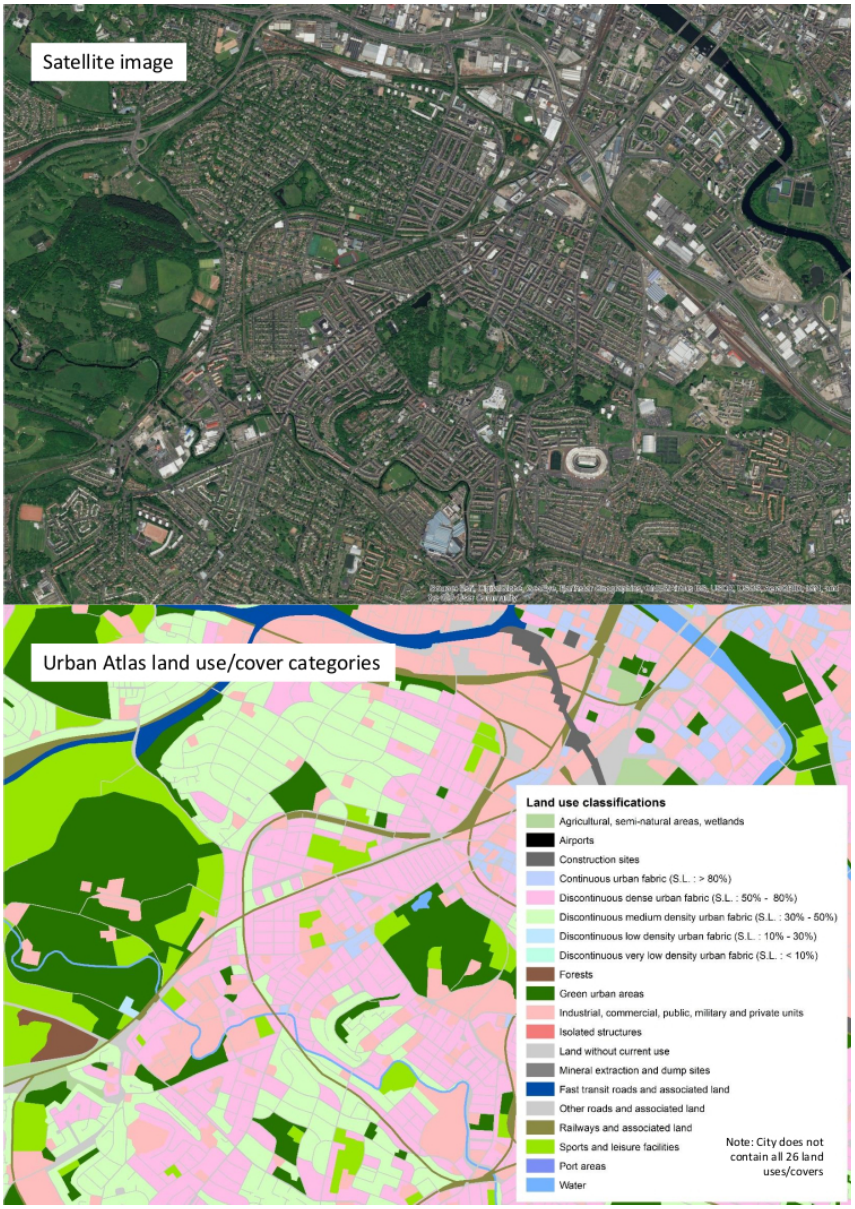

Figure 1: Urban Atlas land use/cover categories and satellite base map.

190x269mm (100 x 100 DPI)

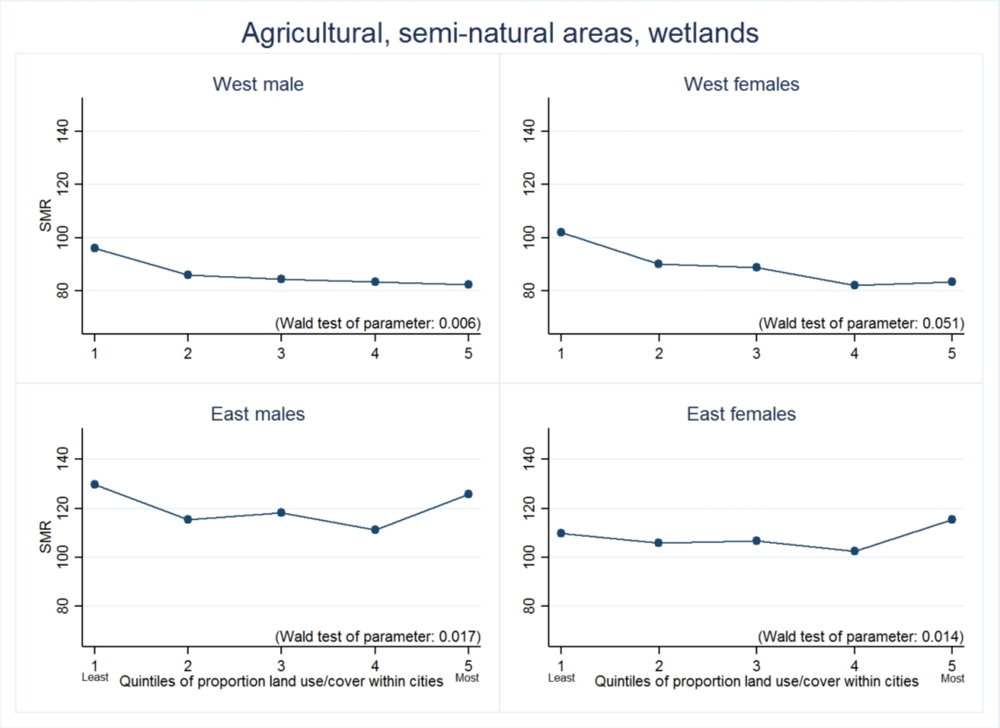

Figure 2: Estimated SMRs for cities with varying quintiles of Agricultural, semi-natural areas, wetlands (quintile 1 = lowest proportion)

385x280mm (96 x 96 DPI)

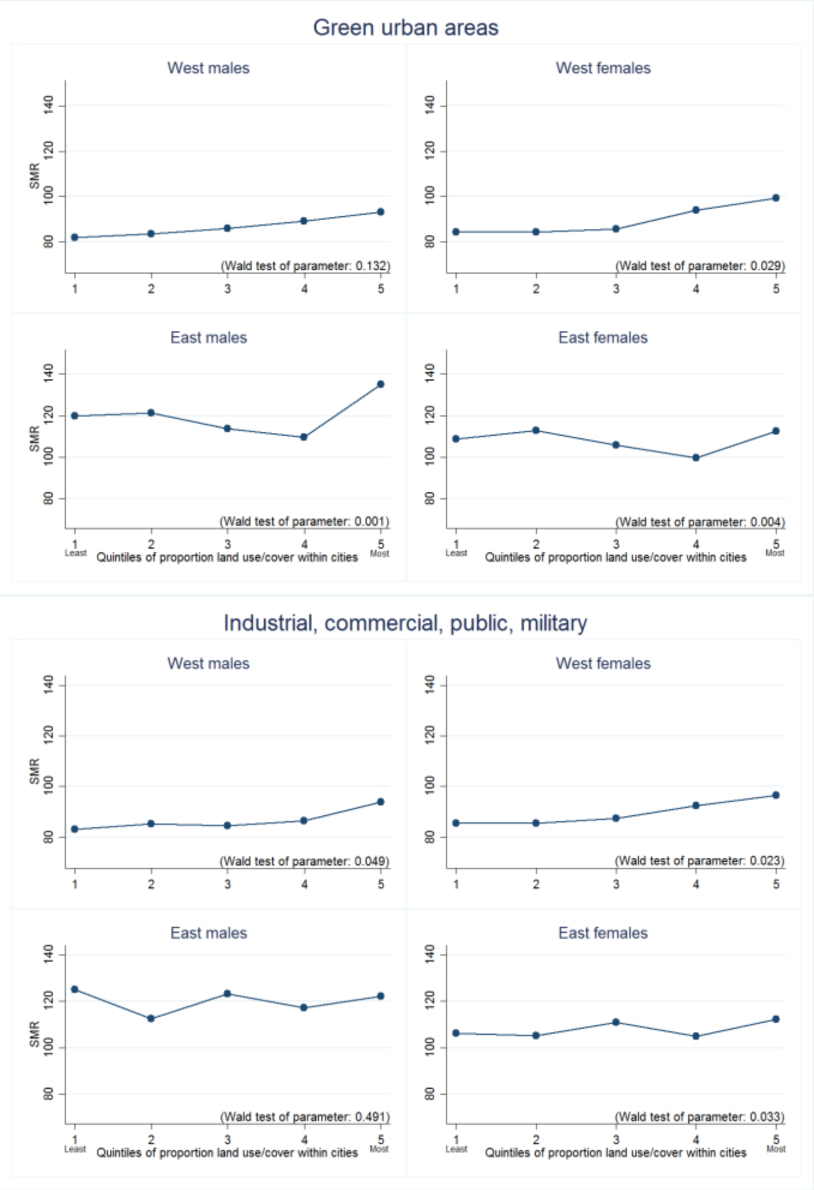

Figure 3: Comparisons between SMR and quintiles land cover/uses for Green urban areas and Industrial, commercial, public, military (partially universal importance by macroregion and/or sex).

173x254mm (100 x 100 DPI)

Which urban land covers/uses are associated with residents' mortality? A cross-sectional, ecological, pan-European study of 233 cities.

Supplementary Table 1: European Urban Atlas land-use definitions.

**1. Artificial surfaces**		Buildings, roads, all constructions of infrastructure and other artificially sealed or paved areas.
**1.1**	**Urban fabric**	Built-up areas and their associated land, such as gardens, parks, planted areas and non-surfaced public areas and the infrastructure, if these areas are not suitable to be mapped separately with regard to the minimum mapping unit size.
1.1.1	Continuous urban fabric	Predominant residential use: areas with a high degree of soil sealing, independent of their housing scheme (single family houses or high rise dwellings, city centre or suburb).
1.1.2	Discontinuous urban fabric	Predominant residential usage. Contains more than 20% non-sealed areas, independent of their housing scheme (single family houses or high-rise dwellings, city centre or suburb). The non-sealed areas might be private gardens or common green areas.
1.1.2.1	Discontinuous dense urban fabric	Residential buildings, roads and other artificially surfaced areas (Average degree of soil sealing: > 50 - 80%).
1.1.2.2	Discontinuous medium density urban fabric	Residential buildings, roads and other artificially surfaced areas. The vegetated areas are predominant, but the land is not dedicated to forestry or agriculture (Average degree of soil sealing: > 30 - 50%).
1.1.2.3	Discontinuous low density urban fabric	Residential buildings, roads and other artificially surfaced areas. The vegetated areas are predominant, but the land is not dedicated to forestry or agriculture (Average degree of soil sealing: 10 - 30%).
1.1.2.4	Discontinuous very low density urban fabric	Residential buildings, roads and other artificially surfaced areas. The vegetated areas are predominant, but the land is not dedicated to forestry or agriculture. Example: exclusive residential areas with large gardens (Average degree of soil sealing: <10 %).
1.1.3	Isolated structures	Isolated artificial structures with a residential component, such as (small) individual farm houses and related buildings. The mapping unit will never be surrounded by any urban class other than transportation network.
**1.2**	**Industrial, commercial, public, military, private and transport units**	At least 30% of the ground is covered by artificial surfaces. More than 50% of those artificial surfaces are occupied by buildings and / or artificial structures with non-residential use, i.e. industrial, commercial or transport related uses are dominant.
1.2.1	Industrial, commercial, public, military and private units	Artificial structures (e.g. buildings) or artificial surfaces (e.g. concrete, asphalt, tar, macadam, tarmac or otherwise stabilised surface, e.g. compacted soil, devoid of vegetation), occupy most of the surface.
1.2.2	Road and rail network and associated land	The road and railway network (COTS navigation data) is ingested into the classification database according to the

For peer review only - http://bmjopen.bmj.com/site/about/guidelines.xhtml

		method given in the Annex within the link noted at the end of this table.
1.2.2.1	Fast transit roads and associated land	Roads defined as "motorways" in the COTS navigation data, and motorway rest and service areas and parking areas, only accessible from the motorways.
1.2.2.2	Other roads and associated land	Roads, crossings, intersections and parking areas, including roundabouts and sealed areas with "road surface".
1.2.2.3	Railways and associated land	Railway facilities including stations, cargo stations and service areas.
1.2.3	Port areas	Administrative area of inland harbours and sea ports. Infrastructure of port areas, including quays, dockyards, transport and storage areas and associated areas.
1.2.4	Airports	Administrative area of airports, mostly fenced. Included are all airport installations: runways, buildings and associated land.
**1.3**	**Mine, dump and construction sites**	Open pit extraction sites (sand, quarries) including water surface. Public, industrial or mine dump sites, raw or liquid wastes, legal or illegal, their protecting dikes and / or vegetation belts and associated land such as service areas.
1.3.3	Construction sites	Spaces under construction or development, soil or bedrock excavations for construction purposes or other earthworks visible in the image.
1.3.4	Land without current use	Areas in the vicinity of artificial surfaces still waiting to be used or re-used.
**1.4**	**Artificial non-agricultural vegetated areas**	Vegetation planted and regularly worked by humans; strongly human-influenced. Sporting facilities as functional units independent of being non-sealed, sealed or built-up.
1.4.1	Green urban areas	Public green areas for predominantly recreational use such as gardens, zoos, parks, castle parks. Suburban natural areas that have become and are managed as urban parks. Forests or green areas extending from the surroundings into urban areas are mapped as green urban areas when at least two sides are bordered by urban areas and structures, and traces of recreational use are visible.
1.4.2	Sports and leisure facilities	All sports and leisure facilities including associated land, whether public or commercially managed: e.g. Theresienwiese (Munich), public arenas for any kind of sports including associated green areas, parking places.
**2. Agricultural + semi-natural + wetland areas**		
2.1	Arable land	Fields under rotation system. Can be non-irrigated or permanently irrigated. Also includes rice fields; Fields laid in fallow are included.
2.2	Permanent crops	Fruit orchards, scattered fruit trees with pasture; Vineyards and their nurseries; Roses; Olive groves; Berries and hop plantations.
2.3	Pasture & natural grassland	Grassland; Pasture and meadow under agricultural use, grazed or mechanically harvested.
2.4	Shrubs and / or herbaceous vegetation	Vegetation cover more than 50%, ground coverage of trees with height > 5 m: < 30%, areas with minor / without

For peer review only - http://bmjopen.bmj.com/site/about/guidelines.xhtml

	including transitional woodland	artificial or agricultural influence; Sclerophyllous vegetation; Bushy sclerophyllous vegetation (e.g. maquis, garrigue); Abandoned arable land with bushes; Woodland degradation: storm, snow, insects or air pollution; Areas under power transmission lines inside forest; Fire breaks; Steep bushy slopes of eroded areas; Abandoned vineyards or orchards, arable land and pasture land under natural colonisation; Dehesas with bush proliferation indicating no agricultural or farming use for a rather long time; Bushy areas along creeks.
2.5	Moors and heathland	Bushes, shrubs and herbaceous plants, dwarf forest in alpine or coastal regions (Pinus Mugo forests). Height is maximum 3 m in climax stage.
2.6	Beaches, dunes, sand	< 10% vegetation cover; Beaches, dunes and sand plains, (coastal or inland location), gravel along rivers; Seasonal rivers, if water is characteristic for a shorter part of the year (< 2 months).
2.7	Bare rocks	> 90% of the land surface of bare rocks, (i.e. < 10% vegetation); Rocks, gravel fields, landslides; Scree (fragments resulting from mechanical and chemical erosion. Weathering rocks forming heaps of coarse debris at the foot of steep slopes), cliffs, rocks.
2.8	Sparsely vegetated areas	Steppes, tundra, badlands, scattered high altitude vegetation. Bare soils inside military training areas. Vegetation cover 10 - 50%.
2.9	Burnt areas	Recently burnt forest or shrubs (but not natural grassland), still mainly black on EO data.
2.10	Snow and ice	Glacier and perpetual snow
2.11	Inland wetlands	Areas flooded or liable to flooding during a large part of the year by fresh, brackish or standing water with specific vegetation coverage made of low shrub, semi-ligneous or herbaceous species; Water fringe vegetation, reed beds of lakes, rivers and brooks. Sedge and fen-sedge beds, swamps; Peat bogs, with or without peat extracting areas; Shallow water areas covered with reed; Seasonal rivers, if water course is not visible in the EO data.
2.12	Coastal wetlands	Areas, flooded or liable to flooding during a large part of the year by brackish or saline water, susceptible to flooding by sea water. Often in the process of filling in and gradually being colonised by halophytic plants; Specific vegetation coverage made of low shrub, semi-ligneous or herbaceous species; Alluvial planes, marshes and intertidal flats; Salinas (salt production sites by evaporation).
**3. Forest (natural and plantation)**		With ground coverage of tree canopy > 30%, tree height > 5 m, including bushes and shrubs at the fringe of the forest; Included are plantations such as Populus plantations, Christmas tree plantations; Forest regeneration / re-colonisation: clear cuts, new forest plantations.

For peer review only - http://bmjopen.bmj.com/site/about/guidelines.xhtml

**4. Water**	The visible water surface area on the EO data is delineated: Sea; Lakes; Fish ponds (natural, artificial); Rivers, including channelled rivers; Canals.

Adapted from: http://ec.europa.eu/regional_policy/sources/tender/pdf/2012066/annexe2.pdf

For peer review only

Which urban land covers/uses are associated with residents' mortality? A cross-sectional, ecological, pan-European study of 233 cities.

Supplementary Table 2: Associations between land cover/use proportion and SMR for Western European cities

Quintiles (proportion of land-use)	Males				Females			
	Coef	p	LL 95% CI	UL 95% CI	Coef	p	LL 95% CI	UL 95% CI
**Agricultural, semi-natural areas, wetlands**								
1 (Least)			ref				ref	
2	-10.22	0.01	-17.69	-2.75	-11.66	0.02	-20.75	-2.58
3	-11.67	0.00	-17.30	-6.04	-13.03	0.02	-23.12	-2.94
4	-12.63	0.00	-19.14	-6.13	-19.71	0.01	-33.78	-5.65
5 (Most)	-13.66	0.00	-21.08	-6.23	-18.32	0.00	-29.77	-6.88
	(Wald test of parameter: 0.006)				*(Wald test of parameter: 0.052)*			
**Airports ***								
1 (Least)			ref				Ref	
2	-2.87	0.29	-8.50	2.76	-3.03	0.46	-11.66	5.61
3	-8.70	0.00	-13.48	-3.93	-10.73	0.02	-19.09	-2.36
4 (Most)	-2.48	0.43	-8.97	4.02	-6.72	0.21	-17.71	4.26
	(Wald test of parameter: 0.005)				*(Wald test of parameter: 0.062)*			
**Construction sites**								
1 (Least)			ref				ref	
2	-3.07	0.37	-10.22	4.07	-9.93	0.13	-23.26	3.40
3	-1.44	0.67	-8.65	5.76	-8.35	0.16	-20.32	3.61
4	-0.68	0.91	-13.98	12.61	-12.36	0.09	-27.14	2.42
5 (Most)	-1.20	0.74	-8.84	6.44	-9.11	0.20	-23.68	5.45
	(Wald test of parameter: 0.814)				*(Wald test of parammeter:0.358)*			
**Continuous urban fabric**								
1 (Least)			ref				ref	
2	1.56	0.60	-4.72	7.84	-5.62	0.10	-12.45	1.21
3	0.00	1.00	-6.03	6.03	-4.66	0.30	-13.98	4.66
4	4.24	0.12	-1.31	9.78	-0.43	0.92	-9.09	8.23
5 (Most)	3.03	0.39	-4.26	10.31	0.09	0.99	-13.58	13.77
	(Wald test of parameter: 0.554)				*(Wald test of parameter: 0.1715)*			
**Discontinuous dense urban fabric**								
1 (Least)			ref				ref	
2	-2.38	0.59	-11.58	6.817	-5.73	0.42	-20.69	9.23
3	-3.39	0.47	-13.27	6.49	-4.45	0.63	-23.80	14.90
4	2.11	0.51	-4.66	8.87	2.28	0.73	-11.88	16.45
5 (Most)	7.36	0.15	-3.02	17.74	9.55	0.26	-7.89	26.99
	(Wald test of parameter: 0.051)				*(Wald test of parameter: 0.093)*			
**Discontinuous medium density urban fabric**								
1 (Least)			ref				ref	

For peer review only

For peer review only - http://bmjopen.bmj.com/site/about/guidelines.xhtml

2	-0.27	0.95	-8.74	8.19	-7.70	0.19	-19.80	4.39
3	-3.20	0.29	-9.40	2.99	-5.60	0.19	-14.34	3.14
4	-4.87	0.10	-10.89	1.16	-11.30	0.04	-21.67	-0.93
5 (Most)	-3.54	0.38	-12.00	4.91	-13.12	0.01	-22.56	-3.67
	(Wald test of parameter: 0.456)				*(Wald test of parameter: 0.097)*			
**Discontinuous low density urban fabric**								
1 (Least)			ref				ref	
2	0.46	0.89	-6.48	7.40	-3.85	0.41	-13.57	5.87
3	-0.40	0.90	-6.95	6.16	-5.65	0.30	-17.07	5.76
4	-0.58	0.92	-12.46	11.31	-6.61	0.50	-27.00	13.79
5 (Most)	7.98	0.01	2.22	13.75	7.92	0.10	-1.62	17.46
	(Wald test of parameter: 0.005)				*(Wald test of parameter: 0.027)*			
**Discontinuous very low density urban fabric**								
1 (Least)			ref				ref	
2	1.10	0.83	-9.74	11.93	-1.87	0.78	-16.24	12.50
3	-5.35	0.25	-14.90	4.212	-13.21	0.10	-29.21	2.79
4	-5.37	0.16	-13.18	2.45	-13.85	0.01	-24.06	-3.65
5 (Most)	-5.75	0.08	-12.28	0.77	-12.42	0.03	-22.99	-1.85
	(Wald test of parameter: 0.313)				*(Wald test of parameter: 0.034)*			
**Residential proportion**								
1 (Least)			ref				ref	
2	1.13	0.74	-5.96	8.22	0.82	0.90	-13.05	14.68
3	2.77	0.29	-2.63	8.17	0.23	0.96	-9.54	10.00
4	3.87	0.08	-0.53	8.26	3.86	0.11	-0.96	8.68
5 (Most)	13.19	0.00	7.88	18.49	17.39	0.00	9.61	25.18
	(Wald test of parameter: <0.001)				*(Wald test of parameter: 0.004)*			
**Fast transit roads and associated land**								
1 (Least)			ref				ref	
2	-5.44	0.24	-14.87	4.00	-14.64	0.03	-27.59	-1.69
3	-3.46	0.25	-9.66	2.74	-10.80	0.03	-20.54	-1.07
4	-3.61	0.41	-12.81	5.59	-15.33	0.02	-28.11	-2.55
5 (Most)	0.62	0.85	-6.44	7.68	-6.77	0.16	-16.47	2.93
	(Wald test of parameter: 0.722)				*(Wald test of parameter: 0.126)*			
**Forests**								
1 (Least)			ref				ref	
2	-4.60	0.11	-10.36	1.158271	-9.71	0.03	-18.44	-0.97842
3	-4.53	0.13	-10.66	1.600225	-8.48	0.10	-18.81	1.854112
4	-9.54	0.06	-19.44	0.372522	-15.13	0.07	-31.88	1.617823
5 (Most)	-9.03	0.03	-16.99	-1.07548	-12.59	0.05	-25.08	-0.10597
	(Wald test of parameter: 0.1562)				*(Wald test of parameter: 0.0048)*			
**Green urban areas**								
1 (Least)			ref				ref	
2	1.60	0.58	-4.47	7.66	-0.02	1.00	-7.11	7.07
3	3.96	0.18	-2.05	9.96	1.43	0.74	-7.81	10.67
4	7.23	0.06	-0.26	14.73	9.57	0.03	1.21	17.93
5 (Most)	11.33	0.02	2.44	20.21	15.06	0.01	4.45	25.67

For peer review only

For peer review only - http://bmjopen.bmj.com/site/about/guidelines.xhtml

	(Wald test of parameter: 0.132)				*(Wald test of parameter: 0.027)*			
**Industrial, commercial, public, military**								
1 (Least)			ref				ref	
2	2.02	0.35	-2.51	6.55	-0.23	0.94	-6.54	6.08
3	1.34	0.67	-5.24	7.93	1.75	0.67	-6.93	10.42
4	3.24	0.22	-2.16	8.65	6.76	0.01	2.31	11.21
5 (Most)	10.64	0.01	3.79	17.50	11.02	0.04	0.93	21.11
	(Wald test of parameter: 0.049)				*(Wald test of parameter: 0.023)*			
**Isolated structures**								
1 (Least)			ref				ref	
2	-5.04	0.16	-12.40	2.32	-9.40	0.07	-19.77	0.97
3	-6.42	0.00	-10.46	-2.39	-10.60	0.02	-18.98	-2.23
4	-7.94	0.02	-14.52	-1.35	-7.23	0.05	-14.35	-0.12
5 (Most)	-7.87	0.04	-15.28	-0.46	-8.46	0.10	-18.68	1.77
	(Wald test of parameter: 0.029)				*(Wald test of parameter: 0.145)*			
**Land without current use**								
1 (Least)			ref				ref	
2	0.65	0.79	-4.57	5.88	-4.16	0.39	-14.34	6.07
3	1.09	0.67	-4.36	6.55	-4.20	0.27	-12.08	3.69
4	0.98	0.67	-3.80	5.77	-1.62	0.43	-5.91	2.67
5 (Most)	10.18	0.05	0.07	20.30	4.58	0.37	-6.12	15.28
	(Wald test of parameter: 0.249)				*(Wald test of parameter: 0.316)*			
**Mineral extraction and dump sites**								
1 (Least)			ref				ref	
2	-3.71	0.22	-9.90	2.47	-6.17	0.11	-13.97	1.63
3	-5.83	0.12	-13.36	1.70	-8.17	0.23	-22.21	5.88
4	-2.74	0.42	-9.85	4.37	-5.45	0.10	-12.10	1.19
5 (Most)	-2.92	0.27	-8.40	2.56	-7.98	0.01	-13.45	-2.52
	(Wald test of parameter: 0.081)				*(Wald test of parameter: 0.061)*			
**Other roads and associated land**								
1 (Least)			ref				ref	
2	-0.95	0.71	-6.38	4.494284	-6.44	0.14	-15.22	2.330186
3	0.64	0.80	-4.66	5.935122	0.46	0.89	-6.43	7.363465
4	2.15	0.39	-3.02	7.322068	4.22	0.15	-1.79	10.23612
5 (Most)	9.49	0.02	1.55	17.42118	6.69	0.17	-3.24	16.61991
	(Wald test of parameter: 0.1889)				*(Wald test of parameter: 0.2091)*			
**Railways and associated land**								
1 (Least)			ref				ref	
2	1.12	0.81	-8.66	10.90	-5.72	0.42	-20.40	8.95
3	2.41	0.31	-2.51	7.31	-2.99	0.66	-17.47	11.49
4	5.82	0.06	-0.21	11.86	-2.74	0.44	-10.10	4.62
5 (Most)	1.81	0.56	-4.71	8.32	-7.73	0.10	-17.10	1.64
	(Wald test of parameter: 0.190)				*(Wald test of parameter: 0.2796)*			
**Sports and leisure facilities**								
1 (Least)			ref				ref	
2	1.68	0.65	-6.25	9.61	-4.11	0.59	-20.19	11.97

For peer review only - http://bmjopen.bmj.com/site/about/guidelines.xhtml

3	2.48	0.36	-3.15	8.12	0.01	1.00	-10.95	10.97
4	4.60	0.06	-0.11	9.31	9.67	0.03	1.18	18.16
5 (Most)	10.39	0.02	1.82	18.95	14.12	0.02	2.88	25.35
	(Wald test of parameter: 0.179)				*(Wald test of parameter: 0.086)*			
**Water bodies**								
1 (Least)			ref				ref	
2	5.33	0.18	-2.70	13.36	6.06	0.45	-10.56	22.69
3	-1.35	0.70	-8.79	6.08	2.29	0.68	-9.55	14.12
4	0.31	0.91	-5.31	5.93	4.27	0.34	-4.96	13.51
5 (Most)	-1.60	0.53	-6.98	3.79	2.32	0.66	-8.86	13.51
	(Wald test of parameter: 0.317)				*(Wald test of parameter: 0.467)*			

* Due to low proportion of land area classed as airports within cities, the first two quintiles combined within the model, essentially representing less than ~0.3% of the total area.

Supplementary Table 3: Associations between land cover/use proportion and SMR for Eastern European cities

Quintiles (proportion of land-use)	Males				Females			
	Coef	p	LL 95% CI	UL 95% CI	Coef	p	LL 95% CI	UL 95% CI
**Agricultural, semi-natural areas, wetlands**								
1 (Least)			ref				ref	
2	-14.45	0.07	-30.34	1.44	-3.68	0.36	-12.24	4.88
3	-11.51	0.20	-30.34	7.32	-2.86	0.44	-10.81	5.08
4	-18.67	0.05	-37.03	-0.32	-7.14	0.13	-16.67	2.39
5 (Most)	-3.89	0.71	-26.56	18.78	5.76	0.39	-8.51	20.03
	(Wald test of parameter: 0.017)				*(Wald test of parameter: 0.014)*			
**Airports ***								
1 (Least)			ref				ref	
2	7.86	0.19	-4.52	20.25	8.08	0.05	0.24	15.92
3 (Most)	15.35	0.02	2.44	28.27	8.64	0.01	2.70	14.58
	(Wald test of parameter: 0.047)				*(Wald test of parameter: 0.008)*			
**Construction sites**								
1 (Least)			ref				ref	
2	0.44	0.94	-11.40	12.27177	-3.87	0.19	-9.93	2.201498
3	1.91	0.66	-7.51	11.33426	0.88	0.80	-6.53	8.280001
4	-4.12	0.52	-17.96	9.71732	-5.57	0.26	-15.84	4.707548
5 (Most)	2.19	0.77	-13.76	18.14004	-4.80	0.24	-13.33	3.721612
	(Wald test of parameter: 0.725)				*(Wald test of parameter: 0.368)*			
**Continuous urban fabric**								
1 (Least)			ref				ref	
2	-1.62	0.78	-14.34	11.10	-0.34	0.92	-7.69	7.01

For peer review only

3	-4.22	0.59	-20.97	12.53	0.40	0.95	-14.96	15.77
4	-6.72	0.39	-23.49	10.05	-0.36	0.96	-15.72	14.99
5 (Most)	-0.93	0.87	-13.68	11.82	5.78	0.36	-7.69	19.25
	(Wald test of parameter: 0.791)				*(Wald test of parameter: 0.070)*			
**Discontinuous dense urban fabric**								
1 (Least)			ref				ref	
2	-3.51	0.51	-15.00	7.98	-0.71	0.89	-11.37	9.96
3	-2.21	0.64	-12.37	7.96	-6.60	0.13	-15.61	2.42
4	-3.91	0.47	-15.38	7.56	-7.30	0.15	-17.71	3.12
5 (Most)	3.36	0.52	-7.86	14.60	-3.33	0.53	-14.60	7.93
	(Wald test of parameter: 0.737)				*(Wald test of parameter: 0.09)*			
**Discontinuous medium density urban fabric**								
1 (Least)			ref				ref	
2	-2.18	0.64	-12.17	7.80	-2.01	0.50	-8.34	4.32
3	7.49	0.08	-1.13	16.12	7.87	0.01	2.48	13.26
4	2.02	0.74	-11.17	15.21	5.07	0.45	-9.24	19.38
5 (Most)	7.92	0.35	-10.01	25.86	1.53	0.80	-11.36	14.42
	(Wald test of parameter: 0.161)				*(Wald test of parameter: 0.083)*			
**Discontinuous low density urban fabric**								
1 (Least)			ref				ref	
2	0.67	0.92	-14.40	15.73	0.19	0.98	-18.09	18.47
3	2.83	0.63	-9.82	15.48	1.37	0.82	-12.03	14.77
4	5.38	0.39	-8.08	18.84	0.88	0.92	-17.35	19.10
5 (Most)	1.47	0.84	-13.93	16.87	-3.82	0.69	-24.54	16.89
	(Wald test of parameter: 0.822)				*(Wald test of parameter: 0.408)*			
**Discontinuous very low density urban fabric**								
1 (Least)			ref				ref	
2	12.10	0.03	1.90	22.30	3.23	0.30	-3.38	9.85
3 (Most)	0.62	0.91	-10.81	12.06	-0.38	0.93	-9.62	8.85
	(Wald test of parameter: 0.065)				*(Wald test of parameter: 0.563)*			
**Residential proportion**								
1 (Least)			ref				ref	
2	-0.70	0.92	-16.68	15.27	-6.19	0.29	-18.52	6.15
3	-4.58	0.40	-16.14	6.97	-2.68	0.64	-14.96	9.60
4	-2.50	0.61	-13.04	8.04	-5.32	0.21	-14.18	3.54
5 (Most)	3.56	0.66	-13.90	21.02	-0.70	0.90	-12.82	11.42
	(Wald test of parameter: 0.469)				*(Wald test of parameter: 0.155)*			
**Fast transit roads and associated land**								
1 (Least)			ref				ref	
2	3.08	0.61	-9.88	16.04	2.18	0.58	-6.31	10.68
3 (Most)	0.83	0.89	-12.17	13.82	2.81	0.63	-9.79	15.41
	(Wald test of parameter: 0.836)				*(Wald test of parameter: 0.846)*			

For peer review only - http://bmjopen.bmj.com/site/about/guidelines.xhtml

**Forests**								
1 (Least)		ref				ref		
2	-6.93	0.28	-20.41	6.55	-13.55	0.02	-24.82	-2.28
3	-5.82	0.13	-13.75	2.11	-10.43	0.00	-16.25	-4.62
4	-0.73	0.89	-12.67	11.21	-8.27	0.13	-19.36	2.83
5 (Most)	-4.99	0.20	-13.08	3.10	-15.96	0.00	-24.74	-7.17
	(Wald test of parameter: 0.309)				*(Wald test of parameter: 0.010)*			
**Green urban areas**								
1 (Least)		ref				ref		
2	1.48	0.67	-6.00	8.95	4.07	0.49	-8.47	16.62
3	-5.99	0.06	-12.37	0.39	-3.06	0.50	-12.89	6.77
4	-10.07	0.05	-19.96	-0.19	-9.26	0.17	-23.29	4.77
5 (Most)	15.37	0.02	3.66	27.08	3.85	0.50	-8.48	16.19
	(Wald test of parameter: 0.001)				*(Wald test of parameter: 0.004)*			
**Industrial, commercial, public, military**								
1 (Least)		ref				ref		
2	-12.53	0.14	-29.98	4.91	-0.92	0.91	-18.26	16.43
3	-1.70	0.81	-16.73	13.32	4.72	0.48	-9.75	19.20
4	-7.70	0.22	-20.69	5.30	-1.34	0.80	-12.77	10.08
5 (Most)	-2.81	0.51	-11.94	6.31	6.09	0.06	-0.19	12.37
	(Wald test of parameter: 0.491)				*(Wald test of parameter: 0.032)*			
**Isolated structures**								
1 (Least)		ref				ref		
2	-5.55	0.38	-19.15	8.04	-7.79	0.15	-18.77	3.19
3	-2.28	0.70	-15.09	10.53	-2.19	0.58	-10.70	6.31
4	-5.12	0.33	-16.15	5.92	-10.66	0.01	-17.95	-3.37
5 (Most)	-6.14	0.33	-19.55	7.26	-12.35	0.00	-16.73	-7.98
	(Wald test of parameter: 0.814)				*(Wald test of parameter: 0.001)*			
**Land without current use**								
1 (Least)		ref				ref		
2	0.14	0.96	-6.55	6.84	-2.02	0.43	-7.45	3.41
3	8.29	0.11	-2.13	18.71	11.30	0.01	3.43	19.18
4	14.75	0.01	5.70	23.81	12.81	0.00	6.49	19.14
5 (Most)	13.67	0.04	1.05	26.29	9.30	0.01	3.09	15.51
	(Wald test of parameter: <0.001)				*(Wald test of parameter: <0.001)*			
**Mineral extraction and dump sites**								
1 (Least)		ref				ref		
2	7.67	0.18	-4.27	19.60	5.02	0.22	-3.42	13.46
3	8.34	0.05	-0.16	16.83	6.73	0.04	0.27	13.18
4	7.86	0.14	-3.11	18.84	7.92	0.12	-2.45	18.30

For peer review only

5 (Most)	7.16	0.22	-4.90	19.23	4.74	0.20	-2.91	12.39
	(Wald test of parameter: 0.139)				*(Wald test of parameter: 0.016)*			
**Other roads and associated land**								
1 (Least)			ref				ref	
2	-8.85	0.07	-18.65	0.94	-1.47	0.61	-7.77	4.82
3	-12.31	0.03	-22.79	-1.82	-4.27	0.22	-11.59	3.05
4	-6.65	0.17	-16.66	3.35	1.69	0.42	-2.76	6.15
5 (Most)	1.52	0.82	-13.31	16.35	7.74	0.15	-3.28	18.76
	(Wald test of parameter: 0.080)				*(Wald test of parameter: 0.174)*			
**Railways and associated land**								
1 (Least)			ref				ref	
2	-3.66	0.43	-13.61	6.29	-1.69	0.61	-8.85	5.46
3	-0.19	0.97	-11.74	11.35	3.45	0.42	-5.74	12.65
4	0.12	0.99	-13.95	14.19	3.57	0.52	-8.36	15.49
5 (Most)	-3.42	0.52	-14.82	7.98	4.93	0.27	-4.39	14.26
	(Wald test of parameter: 0.883)				*(Wald test of parameter: 0.215)*			
**Sports and leisure facilities**								
1 (Least)			ref				ref	
2	-7.42	0.17	-18.66	3.82	-7.20	0.13	-16.97	2.58
3	-18.90	0.00	-27.75	-10.06	-15.15	0.04	-29.78	-0.53
4	-13.80	0.02	-25.27	-2.33	-13.98	0.06	-28.92	0.97
5 (Most)	-19.87	0.00	-31.05	-8.68	-15.16	0.06	-31.38	1.07
	(Wald test of parameter: 0.002)				*(Wald test of parameter: 0.259)*			
**Water bodies**								
1 (Least)			ref				ref	
2	1.97	0.53	-4.69	8.63	0.30	0.91	-5.51	6.10
3	3.49	0.35	-4.42	11.41	1.22	0.70	-5.72	8.16
4	9.52	0.10	-2.14	21.18	5.92	0.02	1.18	10.65
5 (Most)	7.52	0.20	-4.67	19.72	1.55	0.54	-3.90	7.00
	(Wald test of parameter: 0.474)				*(Wald test of parameter: 0.062)*			

* Due to low proportion of land area classed as airports within cities, the first three quintiles combined within the model, essentially representing less than ~0.1% of the total area.

For peer review only

STROBE Statement—Checklist of items that should be included in reports of ***cross-sectional studies***

	Item No	Recommendation
**Title and abstract**	1	(*a*) Indicate the study's design with a commonly used term in the title or the abstract<br>Design stated in study title
		(*b*) Provide in the abstract an informative and balanced summary of what was done and what was found<br>Abstract - Results & Conclusion
**Introduction**		
Background/rationale	2	Explain the scientific background and rationale for the investigation being reported<br>Background - Pages 3-4
Objectives	3	State specific objectives, including any prespecified hypotheses<br>Background – Page 4, lines 15 to 18.
**Methods**		
Study design	4	Present key elements of study design early in the paper<br>Background – Page 4, lines 10 to 14.
Setting	5	Describe the setting, locations, and relevant dates, including periods of recruitment, exposure, follow-up, and data collection<br>Methods, Settings and data sources – Pages 5 to 6
Participants	6	(*a*) Give the eligibility criteria, and the sources and methods of selection of participants<br>Methods, European mortality data (Standardised Mortality Ratios) – Pages 5 to 6
Variables	7	Clearly define all outcomes, exposures, predictors, potential confounders, and effect modifiers. Give diagnostic criteria, if applicable<br>Methods, Statistical analysis – Page 6
Data sources/ measurement	8*	For each variable of interest, give sources of data and details of methods of assessment (measurement). Describe comparability of assessment methods if there is more than one group<br>Methods, Settings and data sources – Pages 5 to 6
Bias	9	Describe any efforts to address potential sources of bias<br>Methods, Statistical analysis – Page 6
Study size	10	Explain how the study size was arrived at<br>Methods, Settings and data sources – Pages 5 to 6
Quantitative variables	11	Explain how quantitative variables were handled in the analyses. If applicable, describe which groupings were chosen and why<br>Methods, Statistical analysis – Page 6
Statistical methods	12	(*a*) Describe all statistical methods, including those used to control for confounding<br>Methods, Statistical analysis – Page 6
		(*b*) Describe any methods used to examine subgroups and interactions<br>Methods, Statistical analysis – Page 6
		(*c*) Explain how missing data were addressed<br>n/a
		(*d*) If applicable, describe analytical methods taking account of sampling strategy<br>Methods, Statistical analysis – Page 6
		(*e*) Describe any sensitivity analyses<br>Methods, Statistical analysis – Page 6

Results

For peer review only - http://bmjopen.bmj.com/site/about/guidelines.xhtml
For peer review only

Participants	13*	(a) Report numbers of individuals at each stage of study—eg numbers potentially eligible, examined for eligibility, confirmed eligible, included in the study, completing follow-up, and analysed	Methods, Settings and data sources – Pages 5 to 6
		(b) Give reasons for non-participation at each stage	n/a secondary ecological analysis
		(c) Consider use of a flow diagram	n/a secondary ecological analysis
Descriptive data	14*	(a) Give characteristics of study participants (eg demographic, clinical, social) and information on exposures and potential confounders	Supplementary tables 2 and 3
		(b) Indicate number of participants with missing data for each variable of interest	n/a
Outcome data	15*	Report numbers of outcome events or summary measures	n/a secondary ecological analysis
Main results	16	(*a*) Give unadjusted estimates and, if applicable, confounder-adjusted estimates and their precision (eg, 95% confidence interval). Make clear which confounders were adjusted for and why they were included	Supplementary tables 2 and 3
		(*b*) Report category boundaries when continuous variables were categorized	n/a secondary ecological analysis
		(*c*) If relevant, consider translating estimates of relative risk into absolute risk for a meaningful time period	n/a secondary ecological analysis
Other analyses	17	Report other analyses done—eg analyses of subgroups and interactions, and sensitivity analyses	Results – Pages 7 to 12
**Discussion**			
Key results	18	Summarise key results with reference to study objectives	Discussion – Page 13, lines 3 to 9
Limitations	19	Discuss limitations of the study, taking into account sources of potential bias or imprecision. Discuss both direction and magnitude of any potential bias	Discussion, Strengths and weaknesses – Pages 15 to 16
Interpretation	20	Give a cautious overall interpretation of results considering objectives, limitations, multiplicity of analyses, results from similar studies, and other relevant evidence	Discussion – Pages 13 to 15
Generalisability	21	Discuss the generalisability (external validity) of the study results	Discussion, Strengths and weaknesses – Pages 15 to 16
**Other information**			
Funding	22	Give the source of funding and the role of the funders for the present study and, if applicable, for the original study on which the present article is based	Funding statement – Page 17

*Give information separately for exposed and unexposed groups.

Note: An Explanation and Elaboration article discusses each checklist item and gives methodological background and published examples of transparent reporting. The STROBE checklist is best used in conjunction with this article (freely

For peer review only - http://bmjopen.bmj.com/site/about/guidelines.xhtml

available on the Web sites of PLoS Medicine at http://www.plosmedicine.org/, Annals of Internal Medicine at http://www.annals.org/, and Epidemiology at http://www.epidem.com/). Information on the STROBE Initiative is available at www.strobe-statement.org.

For peer review only

For peer review only - http://bmjopen.bmj.com/site/about/guidelines.xhtml
